# Transcriptome and network analysis pinpoint ABA and plastid ribosomal proteins as main contributors to salinity tolerance in the rice variety, CSR28

**DOI:** 10.1371/journal.pone.0321181

**Published:** 2025-04-17

**Authors:** Mojdeh Akbarzadeh Lelekami, Mohammad Hadi Pahlevani, Khalil Zaynali Nezhad, Keyvan Mahdavi Mashaki

**Affiliations:** 1 Plant Breeding and Biotechnology Department, Faculty of Plant Production, Gorgan University of Agricultural Sciences and Natural Resources, Gorgan, Iran; 2 Rice Research Institute of Iran, Agricultural Research, Education and Extension Organization (AREEO), Amol, Iran; North Dakota State University, UNITED STATES OF AMERICA

## Abstract

Salinity stress is a major challenge for rice production, especially at seedling stage. To gain comprehensive insight into the molecular mechanisms and potential candidate genes involved in rice salinity stress response, we integrated physiological, transcriptome and network analysis to investigate salinity tolerance in two contrasting rice genotypes. The root and shoot samples were collected at two timepoints (6 hours and 54 hours) of high salt treatment. Element assay showed that the tolerant genotype CSR28 had lower Na^+^/K^+^ ratio in both organs than in those of the sensitive genotype IR28 under salinity stress. A total of 15,483 differentially expressed genes (DEGs) were identified from the RNA-Seq analysis. The salt-specific genes were mainly involved in metabolic processes, response to stimulus, and transporter activity, and were enriched in key metabolic pathways such as, biosynthesis of secondary metabolites, plant hormone signal transduction, and carotenoid biosynthesis. Furthermore, the results showed that the differential genes involved in abscisic acid (ABA) biosynthesis were specifically up-regulated in the tolerant genotype. Network analysis revealed 50 hub genes for the salt-specific genes in the roots of CSR28 which mainly encodes ribosomal proteins (RPs). Functional validation of the nine hub genes revealed three plastid RPs (PRPs), including *OsPRPL17*, *OsPRPS9* and *OsPRPL11*, which contributes to protein synthesis, chloroplast development and stress signaling. Our findings suggested that ABA and PRPs play key roles to enhance of salinity tolerance in CSR28. Our study provides valuable information for further investigations of the candidate genes associated with salt tolerance and the development of salt-tolerant rice varieties.

## Introduction

The world population will reach over nine billion people in 2050; hence, crop production must double to meet the increasing demand for food [1]. Abiotic stresses such as, salinity, drought, heat, and cold, which are exacerbated by climatic changes, are among the most important factors affecting crop production. As one of the major abiotic stresses, salinity influences plant growth and development [2,3]. Approximately 6% (800 million hectares) of the world’s land area is affected by salinity. Furthermore, 20% of cultivable irrigated lands are directly or indirectly affected by salinity [4]. The first symptom of salinity stress appears to be osmotic disorder caused by salt uptake through plant roots, which leads to water deficiency. Long-term stress results in ionic toxicity due to ion imbalance in the cytosol. All of these responses to excessive salinity have detrimental effects on plant productivity. In addition, osmotic stress causes stomatal closure, inhibiting carbon dioxide uptake by plants and leading to reduced photosynthesis. Tolerant plants survive these conditions through a set of compatibility mechanisms involving morphological (like root structure), physiological (such as water regulation), biochemical (like metabolite production) and molecular changes (including gene expression alterations) [5–7]. One of the most important mechanisms is ion homeostasis and reducing the toxic effects of Na^+^. Sodium toxicity is mainly due to its inhibitory effect on enzyme activity and negative effects on metabolism, including the Calvin cycle and other pathways [8]. Moreover, excess Na^+^ in the cytoplasm prevents the absorption and transport of potassium and other mineral elements. Since Na⁺ interferes with K⁺ homeostasis and plays a role in various metabolic processes, maintaining a balanced cytosolic Na⁺/K⁺ ratio has become a crucial mechanism for salinity tolerance [8,9]. Three major groups of genes are involved in ion transport systems. The first group includes salt overly sensitive (SOS) pathway genes that are involved in the sensing of stress and Na^+^ efflux from cells by SOS1. In the second group, Na^+^/H^+^ antiporters (NHX1), are present in the vacuole membrane (tonoplast) and lead to Na^+^ sequestration in vacuoles. Third, high-affinity potassium transporters (HKTs) and salt-sensitive K1 uptake channels (HAKs) are involved in reducing the Na^+^ transition to the shoots and increasing the K^+^ concentration in the cytosol [10,11].

Rice, which has a cultivation area of 168 million hectares and a production of 799 million metric tons per year is considered a staple food for more than half of the world’s population [12]. Rice with a salt tolerance threshold of 3 dS/m is regarded as a very sensitive crop, especially at the seedling and reproductive stages [13–15]. Sodium accumulation in the shoots of rice plays the most destructive role in reducing fertility and yield under long-term salinity treatment [16].

Plant growth and development is regulated by hormones, which also play a role in responding to environmental stresses through signal transduction. Abscisic acid (ABA), as a key hormone, is involved in seed dormancy and germination, senescence, the modulation of root architecture, stomatal regulation and the response to environmental stresses [17]. ABA can also interact with TFs through gene expression to confer tolerance to abiotic stresses, such as, drought and salinity [18].

Ribosomes are large molecular complexes that serve as constituents of protein-synthesizing machinery in all living cells. The cytoplasm, plastids, and mitochondria are the three major sites of protein synthesis in plants. Plastid protein synthesis involves the utilization of a bacterial-type 70S ribosome, which is composed of a small (30S) and a large (50S) ribosomal subunit. The small subunit comprises 16S rRNA and 24 proteins, 12 of which are encoded by plastid genes, while the remaining 12 are encoded by nuclear genes [19]. The large subunit consists of three rRNAs (23S, 5S, and 4.5S) and 33 proteins, of which 8 are encoded by plastid genes and the remaining 25 are encoded by nuclear genes [20]. Ribosomal proteins (RPs) in the large subunit (RPLs) and small subunit (RPSs) of ribosomes play a central role in plant growth and development and in response to environmental stresses [21,22]. The expression of RP-encoding genes is regulated by the interaction of their regulatory sites with phytohormones and stresses. RPL and RPS genes were significantly up-regulated in response to biotic and abiotic stresses [23–25]. The overexpression of RPL14B enhanced drought and salt tolerance in cotton [26]. In transgenic rice, tolerance to drought stress is improved due to overexpression of the RPL23A gene [24]. The Arabidopsis plastid ribosomal protein S5 (PRPS5) is found to be involved in chloroplast development, photosynthesis and resilience to low temperature [27]. However, the specific roles and functions of many RP genes under environmental stresses remain unknown and need to be identified and further studied.

With the advent of genomic technologies such as, next generation sequencing (NGS) and RNA sequencing (RNA-Seq), bioinformatics has become increasingly popular for studying the molecular mechanisms underlying the response to environmental stresses [28]. A powerful analysis within transcriptomics is differential gene expression analysis, which provides methods for studying molecular mechanisms underlying genome regulation and discovering quantitative changes in expression levels between different conditions [29]. Furthermore, another important method for understanding the gene function and gene associations from genome-wide expression is protein-protein interaction (PPI) network analysis, which provides great insight into the functions and interactions of key proteins involved in plant stress tolerance [30,31]. Over the past two decades, several studies have focused on investigating the response of rice to salinity stress through transcriptome analysis [32–37]. However, due to the dynamic nature of gene expression patterns in different genotypes and under different experimental conditions, many molecular aspects of the response to salinity stress remain unclear. Additionally, the relationships between genes and the identification of hub genes involved in the complex process of salinity tolerance are yet to be fully understood.

**Fig 1:**
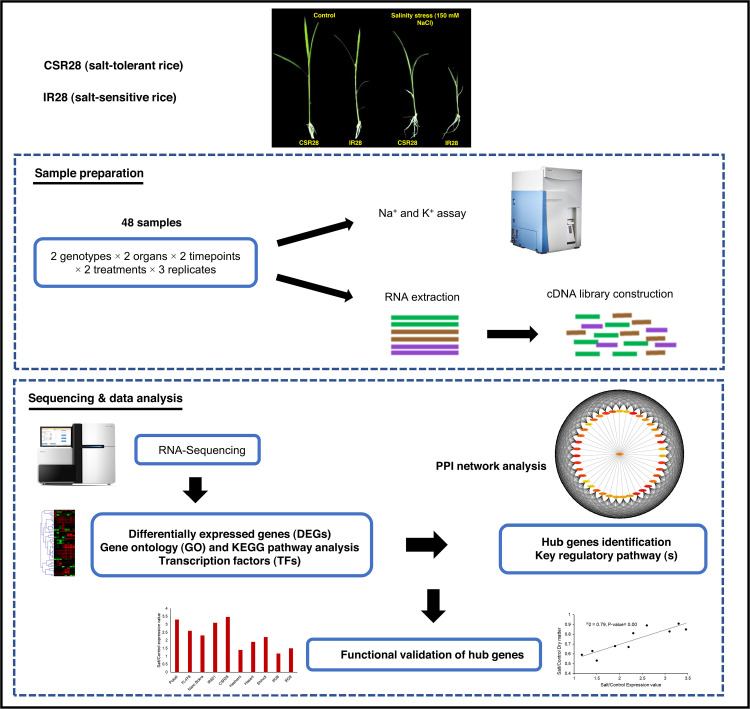
Graphical workflow of experimental design and integration of RNA-Seq and PPI network analysis for identifying key genes and pathways associated with salt tolerance in rice seedling, as well as functional validation of hub genes.

In this study, a comprehensive experiment was conducted using RNA-Seq and network analysis on 48 different datasets generated from two salt-contrasting genotypes, two timepoints, and two organs (Fig 1). Several key TF-encoding genes displayed organ-, timepoint-, and/or genotype-specific salinity stress responses. Gene Ontology (GO) enrichment and KEGG pathway analysis revealed changes in various biological processes and metabolic pathways in response to salinity stress. We identified key genes involved in ABA biosynthesis pathway, secondary metabolite biosynthesis, and ion transporters. Furthermore, network analysis revealed critical hub genes among a vast number of the DEGs. The hub genes were associated with PRPs, and together with the genes encoding ABA biosynthesis pathway, triggered the salinity tolerance of CSR28. This study provides valuable insights for enhancing salt tolerance in rice, contributes to a deeper understanding of the molecular regulatory processes involved in response to salt stress, and establishes a foundation for future advancements in the breeding and genetic improvement of new varieties.

## Materials and methods

### Plant materials and growth conditions

Seeds of two rice (*Oryza sativa* L. ssp. *indica*) genotypes with different salinity tolerances were obtained from the International Rice Research Institute (IRRI) in the Philippines. Salt-sensitive IR28 and salt-tolerant CSR28 (IR51485-AC6534–4) were bred at IRRI, and at the Central Soil Salinity Research Institute (CSSRI) in Karnal, India, respectively. The plants were grown hydroponically in the greenhouse of Heinrich-Heine-University (HHU), Düsseldorf, Germany. First, the seeds were sterilized with 2.5% sodium hypochlorite and, after three rinses they were placed on moistened filter paper in petri dishes for germination. Germination was carried out at 28 °C under dark conditions for 48 hours. The seedlings were transferred to 4-L pots containing Yoshida culture medium [38] and grown under a 14 h light/10 h dark light regime at 28±2 °C. The culture medium was adjusted to pH 5.5 and replaced every three days. Two-week-old seedlings were exposed to 150 mM (15 dS/m) NaCl salinity. The roots and shoots of the control and salt-treated plants were collected at 6 hours and 54 hours after salt exposure.

### Measurement of sodium and potassium contents

Before the sodium and potassium contents were measured, the samples (three replicates of five seedlings each) were washed with distilled water to eliminate salt from tissue surface, and then dried in a 60 °C oven for 4d. The dried tissues were shock-frozen and ground under liquid nitrogen and homogenized overnight in concentrated HNO_3_. The samples were placed in a bain-marie at 95 °C for 30 min, diluted twice with double-distilled water and centrifuged at 4000 rpm for 30 min at 4 ºC and the supernatant was collected and stored at 4 ºC. The sodium and potassium contents of the samples were determined with an iCAP quadrupole ICP-MS (Thermo Fisher Scientific) and normalized based on the sample weight.

### RNA extraction, library construction and Illumina sequencing

RNA extraction was performed by RNeasy Plant Mini Kit (Qiagen, Hilden, Germany) according to the manufacturer’s instruction. To remove DNA contamination, DNase I enzyme treatment was used and RNA quality was measured with Bioanalyzer 2100 (Agilent, Santa Clara, USA). The samples with an RNA integrity number (RIN) ≥ 8 were used to prepare the library. The RNA-Seq library was constructed by TruSeq RNA Sample Preparation Kit (Illumina, San Diego, USA), and the quality and size of cDNA fragments were checked by Bioanalyzer 2100. A total of 48 libraries (two cultivars × two treatments × two organs × two sampling times × three biological replicates) was sequenced by Illumina HiSeq 3000 platform as single-end 150 bp fragments in BMFZ (Biologisch-Medizinisches Forschungszentrum) Genomics and Transcriptomics Laboratory located at Heinrich-Heine-University (HHU). After quality control check of raw sequencing data using FastQC v0.11.7 [39], adapters, primers and low-quality sequences were filtered out by Trimmomatic v0.36 [40]. The sequence reads were submitted to NCBI under GEO accession number PRJNA551583.

### Differentially expressed genes (DEGs) analysis

Tuxedo protocol [41] was used for gene expression analysis. First, high quality reads mapped on the rice IRGSP v1.0 (https://plants.ensembl.org/info/data/ftp/index.html) reference genome using TopHat v2.1.1 [42]. Reference annotation-guided assembly was performed for mapped sequences in all the samples via Cufflinks v2.2.1 [43], and the integrated transcriptome was subsequently obtained via Cuffmerge. Gene expression normalization was performed by calculating the RPKM values. Differentially expressed genes (DEGs) were identified by comparing the gene expression values of different samples using Cuffdiff [44]. The genes with log2 FC (fold change) ≥ 1.5 or log2 FC ≤ −1.5 and FDR (false discovery rate) ≤ 0.05 were considered as significant DEGs. We also grouped the samples based on their expression values using R v4.3.2 software. In this regard, the RPKM (reads per kilobase of transcript per million mapped reads) values were firstly standardized and then hierarchical clustering of the samples was performed based on Euclidean distances and average linkage method. MeV v4.9.0 [45] software was used to display the expression profiles of highly (log10 FC ≥ 3 or log10 FC ≤ −3) DEGs in response to salinity stress (comparisons between control and salinity samples).

### GO and pathway enrichment analysis and identification of transcription factors (TF)

GO enrichment analysis was performed by agriGO database [46] according to Singular Enrichment Analysis (SEA) method with the support of *Oryza sativa* ssp. *japonica* and Rice Gramene Locus background. Hypergeometric statistical test with Hochberg correction (FDR) and P-value cut-off ≤ 0.05 were used to identify significant GO terms. Pathway analysis of the DEGs was performed using Kyoto Encyclopedia of Genes and Genomes (KEGG) database. To identify the TF encoding genes, the DEGs were searched in the Plant Transcription Factor Database (PlantTFDB v4.0) [47] with an E-value cut-off ≤ 10^-5^.

### Quantitative real time PCR (qRT-PCR) analysis

To validate the RNA-Seq results, four genes from the DEGs and the *OsEF1a* housekeeping gene were subjected to quantitative real time PCR (qRT-PCR). The gene specific primers (S1 Table) were designed by Primer Express v3.0 (Applied Biosystems, Foster City, CA). The cDNA was synthesized using LunaScript™ RT SuperMix (NEB, Biolabs, USA) according to the manufacturer’s protocol. The qRT-PCR analysis was performed by StepOnePlus Real-Time PCR System (Applied Biosystem, Foster City, USA) with at least three biological replicates and three technical replicates. The gene expression changes were calculated by the 2^−ΔΔct^ method [48] and R software was used to estimate the correlation between the RNA-Seq and qRT-PCR results.

### Construction of the protein-protein interaction (PPI) network, and identification of hub genes

To gain insights into the functional interactions between the DEGs, we used the STRING online database [49], which integrates known and predicted associations between proteins, including physical interactions and functional associations. A confidence score of 0.4 was used as the cut-off criterion for constructing the PPI networks. The resulting PPI network was visualized using Cytoscape software [50]. To further analyze the network and identify hub genes, we used the CytoHubba plug-in [51] in Cytoscape software. The ranking approach of maximal clique centrality (MCC) in CytoHubba was utilized to identify hub genes. This approach enabled us to identify key genes that may play crucial roles in the biological processes of salt tolerance in rice. The functional enrichment of the hub genes was analyzed using the online database STRING.

### Hub gene validation

To validate the functionality of the identified gene hubs, 10 rice genotypes with a diverse range of salinity tolerances (S2 Table) were selected and grown hydroponically. Two-week-old seedlings were exposed to 150 mM NaCl, and root and shoot samples were collected under control and stress conditions after 54 hours of treatment. For phenotypic evaluation of salinity tolerance, the samples were placed in a 72 °C oven for 48 hours, and salt/control dry weights were calculated for all the genotypes. For gene expression analysis, nine top-interconnected hub genes from the PPI network analysis were chosen, and specific primers (S3 Table) were designed by Primer Express v3.0. All the selected hub genes were belonged to plastid ribosomal proteins. The qRT-PCR analysis was conducted for the root samples under control and salinity conditions, and salt/control expression profiles were calculated for the hub genes in all the genotypes. Linear regression analysis through R software v4.3.2 was performed to determine whether there was a significant relationship (*P* ≤ 0.05) between the expression of any of the hub genes and dry matter.

## Results

### Sodium and potassium contents in roots and shoots

The sodium (Na^+^) and potassium (K^+^) contents and Na^+^/K^+^ ratio of roots and shoots were evaluated by ANOVA and comparison of means (S1 Fig, S4 Table). Increasing the duration of salinity treatment from 6 to 54 hours, led to increased Na^+^ and decreased K^+^ in both organs of both genotypes. Furthermore, the Na^+^/K^+^ ratio increased significantly under the salinity treatment. Although there were no significant differences between the genotypes under the control condition, we observed lower Na^+^, higher K^+^ and lower Na^+^/K^+^ ratios in both organs of CSR28 than in those of IR28 under salinity stress. In particular, salinity stress in the roots resulted in a reduction in the Na^+^ concentration in CSR28 by 20.6% and 40.4% at the 6-hour and 54-hour timepoints, respectively, compared to that in IR28, while the reductions in the shoots were 23.8% and 46%, respectively. Conversely, the absorption of K^+^ in the roots of CSR28 increased 10.4% and 34.3% at 6 hours and 54 hours after salt treatment, respectively, compared to that in the roots of IR28, while the increase in K^+^ in the shoots was 17.2% and 41.4%, respectively (S1 Fig). The Na^+^/K^+^ ratio in both organs significantly differed between the two genotypes only under high salinity, as the ratio in the salt-sensitive group (IR28) was significantly greater than that in the salt-tolerant group (CSR28).

### Global view of gene expression

**Fig 2:**
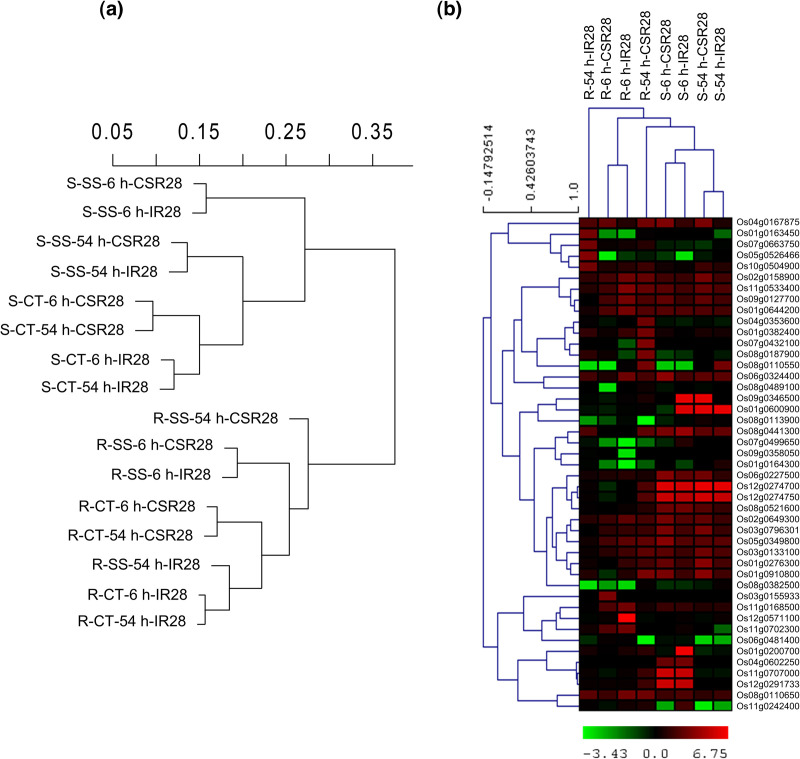
Clustering and heatmap of samples and genes. (a) clustering of samples according to Euclidean distances and Average-linkage method. Data are standardized RPKM values for all genes. (b) Heatmap of 45 highly expressed genes (log10 FC ≥ 3 or log10 FC ≤ -3) in response to salinity stress (comparison of control and salinity samples). Red and green colors indicate increased and decreased expression in response to stress, respectively. R: root, S: shoot, CT: control, SS: salt stress, 6 h: 6-hour timepoint, 54 h: 54-hour timepoint, CSR28: salt-tolerant genotype, IR28: salt-sensitive genotype.

A total of 1.231 billion reads were obtained from all the samples (range 13.51 to 56.06 million) with a total length of 184 Gb. Adapters and low-quality reads (Q ≤ 20) were discarded by Trimmomatic, resulting in 1.204 billion (97.8%) high-quality reads. Among the clean reads, 1.023 billion (84.9%), with an average of 21.31 million per sample, were mapped to the rice reference genome using TopHat2 (S5 Table). The normalized expression values (RPKMs) were used for hierarchical clustering of the samples based on Euclidean distances and average linkage method (Fig 2a). The results showed that the samples clustered into two major groups of the roots and shoots. In the shoots, the control and salinity samples were separated into two subclusters. Under control conditions, the samples were separated according to the genotypes, and for each genotype, the greatest similarity was observed between the timepoints. However, under salinity conditions, the samples were separated by the timepoints and there was the greatest similarity between the genotypes. In the roots, the expression profiles exhibited a more complex pattern, particularly under stress conditions. In contrast to the control conditions and the 6-hour salinity treatment, there was a remarkable difference between the sensitive IR28 and tolerant CSR28 genotypes at the 54-hour timepoint. The expression patterns of the highly expressed genes in the samples in response to salinity stress is displayed in Fig 2b. Most of the correlations between the genotypes were observed in the shoots and at the 6-hour timepoint in the roots, whereas there was a pronounced difference between the roots of IR28 and CSR28 at the 54-hour timepoint. As a result, the gene expression changes in the roots after long-term exposure to 150 mM NaCl were the key factor in differentiating the genotypes in terms of salinity tolerance.

### Identification of differentially expressed genes (DEGs) and transcription factors (TFs)

**Fig 3:**
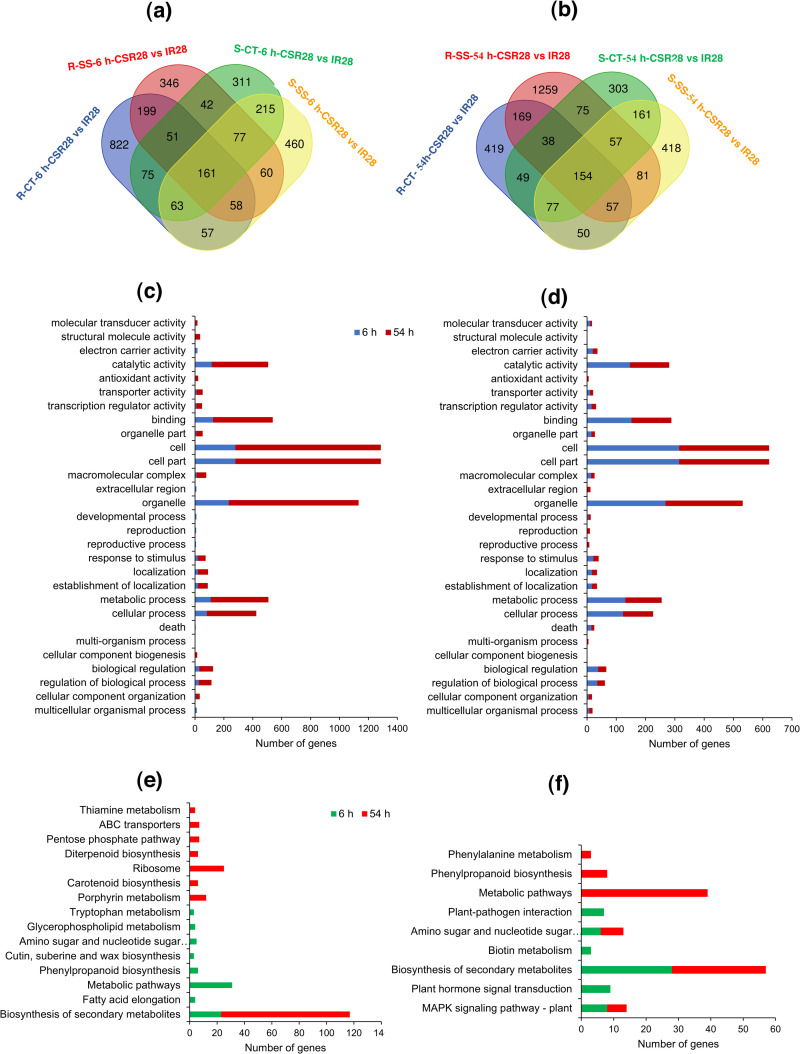
Numbers and functional classification of DEGs. **(a)** and (b) Venn diagram analysis showing specific and common genes for DEGs of 6-h and 54-h timepoints, respectively. R: root, S: shoot, CT: control, SS: salt stress, 6 h: 6-hour timepoint, 54 h: 54-hour timepoint, CSR28: salt-tolerant genotype, IR28: salt-sensitive genotype. **(c)** and (d) Significant GO terms for salt-specific genes in roots and shoots, respectively. **(e)** and (f) Significant KEGG pathways for salt-specific genes in roots and shoots, respectively.

Among the 16 unique experimental samples, Cuffdiff identified a total of 15,483 DEGs according to 32 pairwise comparisons. The highest (7,569 genes) and lowest (29 genes) number were detected in the comparison of the roots and shoots of IR28 at the 6-hour timepoint under control conditions (R-CT-6 h-IR28 vs S-CT-6 h-IR28) and the comparison of two timepoints in the shoots of CSR28 under control condition (S-CT-6 h-CSR28 vs S-CT-54 h-CSR28), respectively (S2 Fig). The comparisons between organs, treatments, genotypes and timepoints is shown in S3 Fig. Under salinity stress at 6-hour timepoint, two genotypes induced almost the same number of DEGs between the roots and shoots (S3a Fig). However, there was a large difference after 54 hours of treatment. Interestingly, 1,283 genes were overexpressed in the roots of CSR28, while only 97 genes were induced in the shoots. This result indicates that the greater salinity tolerance of CSR28 is predominantly due to altered gene expression in the roots. In response to salinity stress, a greater number of differentially expressed genes (DEGs) were detected in the shoots than in the roots (S3b Fig). Specifically, the roots of CSR28 presented a greater number of DEGs (1,540 up-regulated genes and 848 down-regulated genes) as the duration of salinity exposure increased. The DEGs between CSR28 and IR28 is illustrated in S3c Fig. Moreover, under 54 hours of salinity treatment, the roots of CSR28 displayed a significantly greater number of up-regulated genes (1,373) compared to IR28 (521). Conversely, at both timepoints, the shoots of IR28 showed a greater number of up-regulated genes than CSR28. Furthermore, the number of genes that exhibited differential expression between the 6-hour and 54-hour timepoints is depicted in S3d Fig. In both the roots and shoots, the number of DEGs increased in response to salinity stress. Specifically, in the roots, a greater number of genes were induced at the 54-hour timepoint for CSR28, while IR28 had more DEGs at the 6-hour timepoint. In contrast, the shoots of IR28 had a significantly greater number of DEGs at the 54-hour timepoint, and both timepoints in CSR28 exhibited almost the same level of gene induction. In general, increasing the duration of salinity exposure from 6 to 54 hours was associated with an increase in the number of genes expressed in the roots of the salt-tolerant genotype and the shoots of the salt-sensitive genotype. Venn diagram analysis was used to identify specific and common genes between control condition and salinity stress for the genes which were differentially expressed between CSR28 and IR28. At the 6-hour timepoint, 525 and 635 genes were specifically identified under salinity stress in the roots and shoots, respectively (Fig 3a). We also identified 1,472 and 606 genes, which were specifically expressed under salinity stress in the roots and shoots, respectively, at the 54-hour timepoint (Fig 3b). We used these “salt-specific genes” for further analyses.

Using the PlantTFDB, we identified the genes encoding transcription factors that responded to salinity by comparing control and salinity-treated samples. The most abundant transcription factors involved in the response to salinity under different conditions belonged to the WRKY, MYB, bHLH, HB, and AP2-EREBP families (S4a Fig). In the roots of salt-tolerant CSR28, transcription factors belonging to the AP2-EREBP and MYB families were the most important at both timepoints, while in salt-sensitive IR28, the WRKY family was the most abundant. In the shoots and in response to salinity at the 6-hour timepoint, MYB played a greater role in both genotypes, and at the 54-hour timepoint, the AP2-EREBP and MYB families were the most abundant in the tolerant and sensitive genotypes, respectively. The expression levels of genes belonging to the MYB family are shown as a heatmap in S4b Fig. In response to salinity, the expression of various members of the MYB family increased, with the greatest increase observed in the roots of the salt-tolerant genotype at the 54-hour timepoint. While some MYB members, like Os12g0564100 and Os04g0508500, increased in expression across all conditions in response to salinity, others like Os05g0350900 and Os07g0634900 decreased. Additionally, certain genes exhibited specific expression patterns under different conditions. For instance, Os11g0128500 was up-regulated in response to salinity, specifically in the roots of the salt-tolerant genotype, at the 54-hour timepoint.

### Functional classification of salt-specific genes by Gene Ontology (GO) and KEGG pathway enrichment analyses

To functionally classify the salt-specific genes at the three levels of molecular function (MF), cellular component (CC) and biological process (BP), the agriGO database was used. Among the 525 and 1,472 salt-specific genes in the roots, 26 and 21 GO terms were identified at the 6-hour and 54-hour timepoints, respectively (Fig 3c, S6 Table). At the molecular function (MF) level, the terms “binding” (GO:0005488) and “catalytic activity” (GO:0003824) were enriched with the greatest number of genes at both timepoints. However, a larger number of genes were involved at the 54-hour timepoint. The GO terms associated with important molecular responses of plants to environmental stresses, such as, “transcription regulator activity” (GO:0030528), “transporter activity” (GO:0005215), and “antioxidant activity” (GO:0016209), exhibited enrichment with more genes at the 54-hour timepoint.

At the cellular component (CC) level, the terms “cell” (GO:0005623), “cell part” (GO:0044464), and “organelle” (GO:0043226) had the greatest numbers of genes at both timepoints. The terms “organelle part” (GO:0044422) and “macromolecular complex” (GO:0032991) were significantly enriched with more genes at the 54-hour timepoint, while the term “extracellular region” (GO:0005576) was specifically enriched at the 6-hour timepoint.

In terms of biological process (BP), the terms “metabolic process” (GO:0008152) and “cellular process” (GO:0009987) were enriched with the largest number of genes at both timepoints. The functional term “response to stimulus” (GO:0050896) and the regulatory term “biological regulation” (GO:0065007) were significantly more enriched at the 54-hour timepoint. On the other hand, terms related to development and reproduction, such as, “developmental process” (GO:0032502) and “reproduction” (GO:0000003), were specifically involved at the 6-hour timepoint. Overall, it can be concluded that functional terms related to salinity stress in the roots at the 54-hour timepoint played essential roles in differentiating the genotypes for salinity tolerance.

In the shoots, out of the 635 and 606 salt-specific genes, 22 and 27 GO terms were identified at the 6-hour and 54-hour timepoints, respectively (Fig 3d, S6 Table). Similar to the roots, functional groups such as, “catalytic activity”, “binding”, “cell”, “organelle”, “metabolic process”, and “cellular process” were more enriched in the roots than in the shoots. However, unlike those in the roots, the GO terms in the shoots were more enriched at the 6-hour timepoint than at the 54-hour timepoint. Terms associated with stress response, such as, “response to stimulus” and “biological regulation”, were more enriched at the 6-hour timepoint, whereas terms such as “antioxidant activity” at the MF level, “extracellular region” at the CC level, and “reproduction” and “multi-organism process” (GO:0051704) at the BP level were specifically involved at the 54-hour timepoint. A portion of the co-functional network of BP-related GO terms associated with salt-specific genes in the roots at the 54-hour timepoint is displayed in S5 Fig. This network revealed significant enrichment of functional groups such as, “response to stimulus” and “ion transport” (GO:0006811). Ion transporters are crucial proteins for sodium detoxification and the maintenance of ion balance under salinity stress in roots.

To identify the pathways of molecular and metabolic processes, a pathway analysis of salt-specific genes was conducted using the KEGG database (Fig 3e and 3f). The pathway of biosynthesis of secondary metabolites (ko01110), was found to be highly enriched in both organs and timepoints. Among the genes involved in the biosynthesis of secondary metabolites, 26 genes exhibited high expression in one of the two genotypes (S6 Fig). In the roots at the 54-hour timepoint, a greater number of genes were expressed in the tolerant genotype than in the sensitive genotype. Terpenes, flavonoids, phenylpropanoids, and wax compounds were associated with the greatest number of genes. The Genes Os02g0484200 (*OsMaT-1*, involved in the biosynthesis of flavonoids), Os04g0638401 (related to flavonoid biosynthesis), and Os07g0464200 (involved in phenylpropanoid biosynthesis) exhibited increased expression under all conditions in the tolerant genotype. Conversely, Os04g0481800 (associated with wax compound biosynthesis) exhibited greater expression in the sensitive genotype across all conditions.

We also observed different functional pathways involved in the timepoints of the roots. Metabolic pathways (ko01100), phenylpropanoid biosynthesis (ko00940), amino sugar and nucleotide sugar metabolism (ko00520), fatty acid elongation (ko00062), and others were identified at the 6-hour timepoint, while ribosome (ko03010), porphyrin metabolism (ko00860), ABC transporters (ko02010), Carotenoid biosynthesis (ko00906), and other pathways were involved at the 54-hour timepoint. In contrast, the shoots represented almost the same KEGG pathways at both timepoints. In addition to the biosynthesis of secondary metabolites, the MAPK signaling pathway – plant (ko04016) and amino sugar and nucleotide sugar metabolism (ko00520) were commonly enriched at the 6-hour and 54-hour timepoints, while plant hormone signal transduction (ko04075), plant‒-pathogen interaction (ko04626) and biotin metabolism (ko00780), which were specifically observed at the 6-hour timepoint, and metabolic pathways, phenylpropanoid biosynthesis and phenylalanine metabolism (ko00360) were specifically enriched at the 54-hour timepoint.

### Validation of differential gene expression by qRT-PCR

The expression values of the four selected genes in response to salinity stress are shown in S7a Fig, indicating a similar pattern between the RNA-seq and qRT-PCR approaches. The sequencing data were strongly correlated (R^2^=0.85) with the expression values of qRT-PCR analysis (S7b Fig).

### PPI-based network analysis revealed hub genes across the salt-specific genes

**Fig 4:**
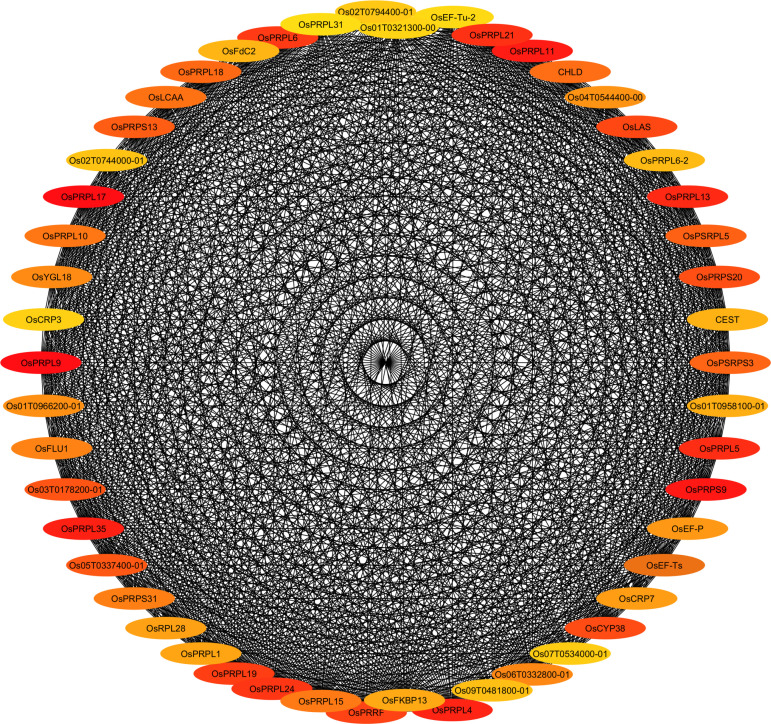
Protein-protein interaction (PPI) network analysis for up-regulated salt-specific genes in the roots of salt-tolerant CSR28 at 54-hour timepoint. Top 50 hub genes are indicated by color intensity. Red nodes display highly dens interactions with other proteins.

Protein-protein interaction (PPI) network analysis was employed to explore the associations between the salt-responsive genes. Out of the 1,472 DEGs identified between the genotypes in the roots at the 54-hour timepoint, 1,130 and 342 genes were up-regulated in CSR28 and IR28, respectively. Among the 1,130 up-regulated genes in CSR28, 50 hub genes were identified on the basis of the Cytohubba plugin in Cytoscape, including OS02T0822600-01, OsJ_13095, OsJ_36392, RPS9, OS03T0122200-01, OsJ_22158, OS03T0265400-01, OsJ_03455, RPL5, OsJ_06142 and so on (Fig 4). Among the 342 up-regulated genes in IR28, 25 hub genes with lower interconnected scores were identified, such as OsJ_00707, CYCB2–2, OsJ_32048, OsJ_09263, KIN10A and OS01T0931200-01 (S8 Fig).

### Functional classification of hub genes

The GO enrichment analysis of the hub genes using STRING revealed that GO terms such as, “translation”, “cellular nitrogen compound biosynthetic process” and “gene expression” at the BP level, “rRNA binding” and “structural constituent of ribosome” at the MF level and “chloroplast” and “ribosome” at the CC level were significantly involved in CSR28 (S9 Fig, S7 Table). In IR28, the GO terms such as, “cell division” and “microtubule-based process” at the BP level, “microtubule binding” and “cytoskeletal protein binding” at the MF level and “microtubule cytoskeleton”, “supramolecular complex” and “spindle” at the CC level had the highest levels of significance (S9 Fig, S8 Table). KEGG pathway analysis revealed that “ribosome” was the only pathway enriched for the hub genes of CSR28. Of the 50 hub genes in CSR28, 21 genes were significantly involved in the “ribosome” KEGG pathway (S9 Table). No KEGG pathways were associated with the hub genes of IR28 roots at the 54-hour timepoint.

### Functional validation of the hub genes

**Fig 5:**
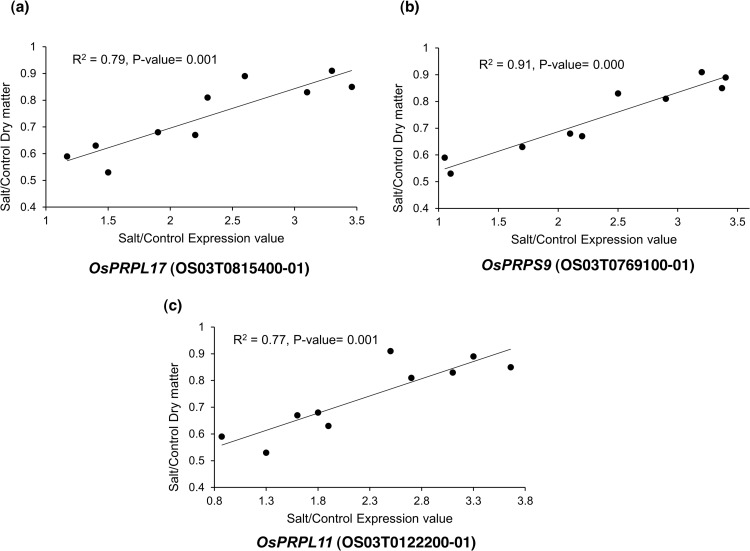
Functional validation of three hub genes encoding plastid RPs in response to salinity stress. A significant relationship was shown between dry weight and expression value of three hub genes, including *OsPRPL17* (R^2^= 0.79, P-value= 0.001), *OsPRPS9* (R^2^= 0.91, P-value= 0.000) and *OsPRPL11* (R^2^= 0.77, P-value= 0.001).

We selected nine top-interconnected hub genes (red nodes) from the PPI network analysis (Fig 4, Table 1) to test the correlation between the salinity tolerance of various rice genotypes and their corresponding expression profiles for any of the hub genes. The results showed that the expression profiles of three plastid RP-encoding genes, namely, *OsPRPL17* (OS03T0815400-01), *OsPRPS9* (OS03T0769100-01) and *OsPRPL11* (OS03T0122200-01), were significantly related to their corresponding dry matter in response to salinity stress (Fig 5), whereas six other plastid RP-encoding genes, namely, *OsPRPL9* (OS02T0822600-01), *OsPRPL35* (OS06T0647100-01), *OsPRPL4* (OS03T0265400-01), *OsPRPL13* (OS01T0749200-01), *OsPRPL5* (OS03T0125000-01) and *OsPRPL21* (OS02T0259600-01) were not functionally validated (S10 Fig).

**Table 1:** Top-interconnected hub genes from PPI network analysis encoding plastid RPs in the roots of CSR28 at 54-h timepoint.

Gene symbol	Transcript ID	Arabidopsis ortholog (E-value)	Putative function	Reference
*OsPRPL9*	OS02T0822600-01	AT3G44890.1 (7e-51)	50S ribosomal protein L9	[35,52]
*OsPRPL17*	OS03T0815400-01	AT3G54210.1 (5e-60)	50S ribosomal protein L17	[53]
*OsPRPS9*	OS03T0769100-01	AT1G74970.1 (5e-54)	30S ribosomal protein S9, Early chloroplast development	[54,55]
*OsPRPL11*	OS03T0122200-01	AT1G32990.1 (2e-83)	50S ribosomal protein L11	[56,57]
*OsPRPL35*	OS06T0647100-01	AT2G24090.1 (2e-37)	50S ribosomal protein L35, chloroplast precursor (CL35)	[52]
*OsPRPL4*	OS03T0265400-01	AT1G07320.4 (6e-42)	50S ribosomal protein L4, chloroplast precursor (R-protein L4)	[58]
*OsPRPL13*	OS01T0749200-01	AT1G78630.1 (1e-100)	50S ribosome L13 protein, Chloroplast development under low temperature conditions	[59]
*OsPRPL5*	OS03T0125000-01	AT4G01310.1 (6e-29)	50S ribosomal protein L5, chloroplast	[58]
*OsPRPL21*	OS02T0259600-01	AT1G35680.1 (3e-44)	50S ribosomal protein L21, chloroplast precursor (CL21) (CS-L7)	[60]

The hub genes in bold were validated in functional validation experiments.

## Discussion

Plant cells normally contain 100–200 mM of K^+^, 1–10 mM of Na^+^ and a Na^+^/K^+^ ratio of 0.01–0.05. The high accumulation of Na^+^ under NaCl stress prevents the absorption of essential K^+^ and consequently disrupts the typical enzymatic and metabolic functions of cells. Therefore, maintaining a low concentration of Na^+^ or a low Na^+^/K^+^ ratio in the cytosol is vital for cell functioning [61,62]. In both genotypes in the present study, Na^+^ increased, K^+^ decreased, and the Na^+^/K^+^ ratio increased in response to salinity (S1 Fig). The roots, as the first vital barrier against salinity stress, had greater concentrations of Na^+^ as compared to the shoots. After long-term salinity treatment, Na^+^ absorption increased in both organs. The greater difference between the genotypes after 54 hours of salinity treatment suggests that more mechanisms are involved in preventing the absorption of toxic ions in CSR28, for instance, ion exclusion of root cells over time. Tolerant varieties can absorb more K^+^ and prevent Na^+^ accumulation to maintain a low Na^+^/K^+^ ratio. The roots play key roles in protecting plants against excessive absorption of salt, excluding salt from the root environment, returning that salt to the soil and absorbing water [4]. On the other hand, the difference between the Na^+^ content in the shoots of the genotypes was greater than that in the roots, indicating a lower transfer of Na^+^ to the shoots of the salt-tolerant genotype. Conversely, both organs of CSR28 absorbed more K^+^, and less Na^+^ compared to IR28 under salinity treatment, and increasing its duration, led to a decreased Na^+^/K^+^ ratio.

In the present study, RNA-seq was utilized to sequence 48 samples obtained from the roots and shoots of two rice contrasting genotypes, under control and high salinity stress at two 6-hour and 54-hour sampling times in three biological replicates. Approximately 84.9% of the cleaned reads were mapped to the rice reference genome (IRGSP v1.0), suggesting alignment to exonic regions. A hierarchical clustering of Pearson’s correlation in the transcriptome data revealed patterns of gene expression across different samples. Venn diagram analysis of the DEGs showed that after 6 hours of salinity treatment, 525 and 635 and genes were differentially induced between the genotypes in the roots and shoots, respectively. Moreover, at the 54-hour timepoint, 1,472 and 606 genes were involved in the roots and shoots, respectively. These salt-specific genes were employed for further analysis. In general, our findings indicated that long-term salinity stress in the roots was able to distinguish the CSR28 and IR28 genotypes in terms of salt tolerance. Several reports have shown that the molecular mechanisms of roots are responsible for inducing salt tolerance in rice seedlings [10,32,36,63]. The GO enrichment analysis of the salt-specific genes in the roots at the 54-hour timepoint, revealed important terms such as, “ion transport” (Fig 2, Fig 3a and 3b, S3 Fig).

The expression of stress-responsive genes under abiotic stresses is regulated through several transcription factors (TFs) such as, MYB, bZIP, WRKY, AP2/ERF, and bHLH [64–67]. Overall, TFs bind to specific elements called cis-elements in the promoter regions of downstream functional genes, and modulate their expression levels. Therefore, they play a key role as regulators of tolerance to abiotic stresses [68,69]. Several important genes encoding transcription factors were identified in this study using the PlantTFDB (S4 Fig). The *OsMYB6* gene (Os04g0676700) was identified as one of the most important members of the MYB family in the shoots of both genotypes in response to elevated salinity. The overexpression of this gene plays a major role in inducing tolerance to salinity and drought in transgenic rice [70]. The *OsNAC14* gene (Os01g0675800) was up-regulated in response to salinity in both genotypes under all conditions. This gene belongs to the NAC family and was reported to be induced in response to drought by [71]. Two genes from the AP2/ERF family, *OsDREB1G* (Os02g0677300) and *OsERF57* (Os07g0227600), were induced in the roots of CSR28 at the 54-hour timepoint. Since these genes have ethylene-responsive domains, they are likely to be major components of the ethylene-signaling pathway. The *OsWRKY80* (Os03g0855100) and *OsWRKY40* (Os11g0117500) genes, which are members of the WRKY family, were induced in salt-tolerant CSR28. *OsWRKY80* is induced in response to rice blast disease, and its expression was up-regulated through the external application of jasmonic acid and ethylene [72]. Compared with those of IR28, the expression of the *OsbHLH1* gene (Os01g0928000), which is a member of the bHLH family, was elevated only in the roots of CSR28 at 54 hours. Previous investigations indicated that the overexpression of *OsbHLH1* helps to maintain ionic balance and improve salt tolerance in rice through the induction of AKT1 [73]. The contrasting genotypes were significantly different in terms of the MYB members induced in the roots and long-term exposure to salinity. Among the 10 identified genes, eight (including Os12g0564100, Os01g0589900, Os07g0191500, Os05g0574800, Os07g0629000, Os02g0529900, Os09g0414300, and Os01g0182400) were overexpressed in salt-tolerant CSR28 compared to those in IR28 (S4b Fig). These novel genes may play a key role in inducing salinity tolerance in CSR28. The specific expression of the identified transcription factors in this study can be utilized in marker-assisted selection programs.

Based on the KEGG pathway analysis, salt-specific genes were found to be involved mainly in metabolic pathways, biosynthesis of secondary metabolites, carotenoid biosynthesis, porphyrin metabolism, and plant hormone signal transduction (Fig 3e and 3f). These annotations provide a valuable resource for identifying specific biological processes, pathways, and molecular functions underlying salt stress tolerance in rice.

Among the genes identified in the biosynthesis of secondary metabolites (S6 Fig), three key genes were involved in tolerance to environmental stresses. The gene *OsGL1–6*; Glossy1-homologous gene 6 (Os02g0814200), a member of the fatty aldehyde decarbonylase gene family, is homologous to the CER1 gene in Arabidopsis, and has been reported to play a role in the biosynthesis of leaf wax compounds and consequently drought resistance in rice [74]. Our findings also revealed a key gene involved in terpene biosynthesis, including *OsTPS10*; Terpene synthase 10 (Os03g0348200). Terpenes are a large group of secondary metabolites produced in response to biotic and abiotic stresses. The gene *OsTPS20* in rice is involved in terpene production in response to oxidative stress [75]. The gene *OsCAD3*; Cinnamyl alcohol dehydrogenase (Os10g0430200), was expressed specifically in the roots of CSR28 at the 54-hour timepoint. The CAD genes are involved in the final step of the phenylpropanoid biosynthesis pathway, and 12 genes from this family have been reported in rice [76]. Among these genes, *OsCAD3* has been found to be a key gene involved in the response to environmental stresses [77].

ABA signaling pathway plays a key role in regulating abiotic stress in plants. Fauzia et al. [11] reported that the pathway of carotenoid biosynthesis was significantly involved in the salt-tolerant Japonica rice, SZK. In the present study, all six genes involved in carotenoid biosynthesis in the roots at the 54-hour timepoint, including *OsBCH2* (Os04g0578400), *OsPSY2* (Os12g0626400), *OsCYP97C2* (Os10g0546600), *OsPSY1* (Os06g0729000), *OsRVDE1* (Os04g0379700) and *OsABA8OX3* (Os09g0457100), were remarkably up-regulated in salt-tolerant CSR28 compared to salt-sensitive IR28 (S11 Fig). Zhang et al. [78] reported that *OsASR6*, which is expressed by ABA, stress and ripening, enhances salt tolerance in rice.

Despite significant efforts to understand the molecular mechanisms of salt tolerance in rice, these mechanisms remain unknown. The interpretation of biological datasets has become more complex with the emergence of transcriptome profiling experiments. To fully exploit the potential of transcriptome data, novel system-level analyses are needed to uncover significant correlations between genes and biological processes, as well as the regulatory mechanisms governing particular responses [79]. Biological networks have become a popular and effective way of illustrating the intricate organization of biological systems and deciphering the complex relationships between genes [80]. Hence, in the present study, we used the STRING database to construct a PPI network of the salt-specific genes and employed the CytoHubba plugin of Cytoscape software to identify hub genes. The results of network analysis of the roots at the 54-hour timepoint, demonstrated a more significantly interconnected network of the salt-tolerant genotype CSR28 than of the salt-sensitive genotype IR28 (Fig 4, S8 Fig). The functional GO and pathway analysis of the top 50 hub genes identified in CSR28 revealed the biological process associated with protein-synthesizing machinery, while the top 25 hub genes identified in IR28 were involved mainly in the cell cycle process. Due to the potential impact of salt stress on protein synthesis, it has been shown that under stressed conditions in plants, the up-regulation of genes encoding RPs can lead to more effective reconstruction of the cellular protein-synthesizing machinery [57,81]. For instance, in salt-tolerant Pokkali rice, numerous RPs such as, RPS4, 7, 8, 9, 10, 19, 26, RPL2, 5, 18, and 44 were significantly up-regulated during salt stress [82]. On the other hand, protein synthesis requires a high concentration of K^+^ to effectively bind tRNA to ribosomes [11,83]. In the present study, the elevated concentration of K^+^, along with the up-regulation of genes encoding RPs under salt stress conditions, suggested a reduction in damages to the translation process and a mechanism of salinity tolerance in the tolerant genotype.

Further, the PPI network analysis displayed that plastid ribosomal proteins (PRPs) were strongly interconnected with other proteins. Therefore, these hub genes were subjected to functional validation analysis. Among the nine selected hub genes, three PRPs including *OsPRPL17*, *OsPRPS9*, and *OsPRPL11* exhibited a significant regression relationship with their corresponding dry matter in response to salinity stress (Fig 5). Barratt et al. [84] identified three validated hub genes for early thermotolerance in wheat through a regression analysis between the hub gene RPKM and normalized dry biomass loss.

In accordance with our findings, *OsPRPL17*, which encodes the plastid ribosomal large subunit protein L17, has been shown to be up-regulated in tolerant cultivar of rice in response to long-term drought [53]. Since under long-term salinity conditions, the plants suffer from dryness, leaf senescence and reduced photosynthetic ability, we hypothesized that *OsPRPL17* was effective at compensating for the damage caused by water scarcity through the synthesis of RPs and the restoration of photosynthetic activity. The down-regulation of many genes encoding proteins involved in the protein synthesis process has been reported in sensitive rice cultivars under drought stress, whereas this down-regulation was much less pronounced in tolerant rice cultivars [53].

*OsPRPS9*, encoding the plastid ribosomal small subunit protein S9, is found to be up-regulated in abiotic stresses such as salinity, drought and heat, suggesting that it is a core abiotic stress-responsive gene [54]. Generally, we hypothesized that RPs play key roles in multiple environmental stresses due to their highly dense interactions. Furthermore, *OsPRPS9* co-localized with stress-tolerance QTL regions, including qDT5 [85] and qSBR-3 [86], for drought tolerance and sheath blight resistance, respectively. This RP is also involved in chloroplast development. A defective form of *OsPRPS9* (*wgl2* mutant) led to an albino phenotype with abnormal chloroplasts and lower levels of photosynthetic pigments [55].

Another validated hub gene identified in the present study was *OsPRPL11*, which encodes the plastid ribosomal large subunit protein L11. An ortholog of PRPL11 in Arabidopsis (AT1G32990.1) has been reported to be up-regulated under salinity stress. A knockout mutant deficient in PRPL11 (prpl11) exhibited enhanced sensitivity to salinity stress due to reduced translation of the large subunit of Rubisco (RbcL), a CO2-fixing enzyme located in the plastid stroma, severe growth depression and pale leaves [57,87]. These findings indicated the importance of PRPL11 for protein synthesis in plastids and normal photosynthetic functions of chloroplasts in response to salinity stress. As a result, the accumulation of plastid ribosomal proteins in the chloroplasts of CSR28, enabled the plants to maintain their photosynthetic capacity and greater biomass level than the sensitive genotype IR28 under salinity stress. These validated hub genes can be served as biomarker for selection of salt-tolerant rice genotypes in breeding programs.

Although, plant RPs play universal roles in translation, they are also involved in hormone signaling pathways [88]. Considering that the expression of genes involved in carotenoid biosynthesis (S11 Fig) leads to the production of abscisic acid (ABA), we believe that the RPs identified in this study may play a crucial role in sensing and transducing stress signals by interacting with ABA, and consequently, enhancing the salt tolerance of CSR28. It has been reported that up-regulation of the ribosomal protein L6, RPL6 improved salt tolerance in transgenic rice through enhancing the expression of proteins associated with growth, development and signal transduction pathways [89]. It has been shown that RPL10A is triggered by ABA and may serve as a positive regulator for ABA-related responses in Arabidopsis plants [88].

In addition to the RPs, the network analysis revealed a chloroplast protein enhancing stress tolerance (CEST), which confers tolerance to multiple environmental stresses and reduces photooxidative damage in transgenic Arabidopsis [90].

**Fig 6:**
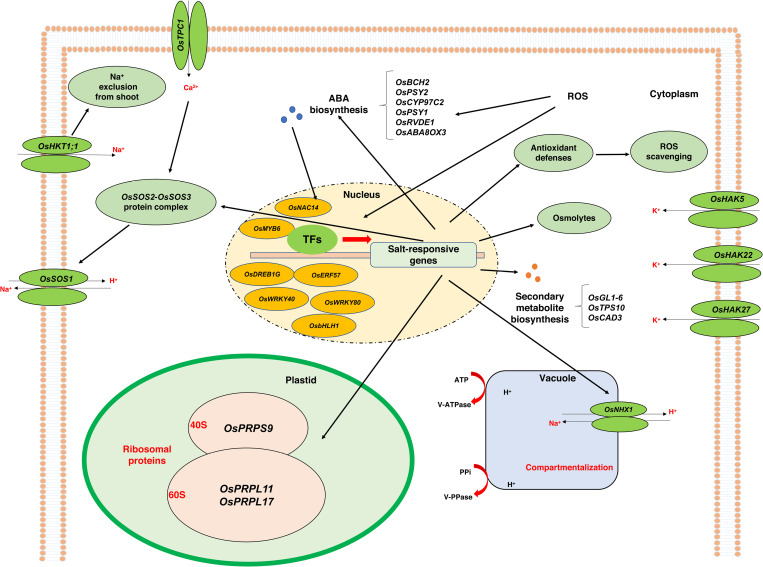
Molecular mechanism of rice salt tolerance according to the critical genes identified in the present study. Salt-induced elevation of signaling molecules activated transcriptional factors followed by several key genes involved in ABA biosynthesis, secondary metabolite biosynthesis, ribosomal proteins, osmolytes and ion transporters, and consequently resulted to Na^+^ detoxification, ion homeostasis and osmotic adjustment in the cells.

The molecular mechanism of rice salt tolerance based on the key genes identified in this study is illustrated in Fig 6. Molecules and stress sensors such as Ca^+2^ and ROS transmit stress signals to activate transcription factors and lead to the expression of salt-responsive genes. The overexpression of genes involved in ABA biosynthesis, secondary metabolite biosynthesis, ribosomal proteins, osmolytes, antioxidant compounds, and ion transporters resulted in better sensing of salt stress, enhanced photosynthetic capacity, osmotic regulation, ROS and Na^+^ detoxification, and osmotic balance under harsh stress conditions. The *OsTPC1* gene, found in the plasma membrane, plays a crucial role in salinity tolerance by facilitating the passage of Ca^+2^, which acts as a secondary messenger in plant signal transduction pathways under various stressors [91]. High-affinity potassium transporters (HKT1 genes) such as *OsHKT1;1* and *OsHKT1;4* are essential for maintaining ion homeostasis and preventing the transport of toxic Na^+^ to shoots in the roots of salt-tolerant plants [92,93]. The transporters such as *OsSOS1*, *OsNHX1*, and *OsHAKs* also contribute to ion homeostasis and salt tolerance by regulating Na^+^ and K^+^ concentrations in different plant organs. The differential expression of these genes between salt-tolerant and salt-sensitive genotypes, especially at specific timepoints, underscores their importance in responding to and coping with salt stress.

## Conclusions

In conclusion, the study of the transcriptome dynamics of two contrasting rice genotypes under salinity stress using RNA-Seq led to the identification of 15,483 differentially expressed genes (DEGs), including salt-specific genes involved in metabolic processes, response to stimulus, and transporter activity. Protein-protein interaction (PPI) network analysis revealed that the hub genes, which were mainly involved in ribosomes and encoding RPs, may play a role in stress signaling and tolerance enhancement in the salt-tolerant genotype. Our findings revealed a potential interaction between abscisic acid (ABA) and RPs for stress signaling. Furthermore, the functional validation analysis revealed three key RPs including, *OsPRPL11*, *OsPRPL17* and *OsPRPS9*, through a significant regression analysis between salt tolerance-related phenotypic traits and gene expression values. The results of the element assay and identification of ion transporters indicated that salt-tolerant CSR28 maintained a low Na^+^/K^+^ ratio in the shoots under high salinity stress. Our findings also indicated that the higher concentration of K^+^ and up-regulation of genes encoding RPs under salt stress conditions lead to reduced damage in the translation process and restoration of photosynthetic activities. Further, key transcription factors (TFs) with specific expression patterns were identified, providing valuable information for further investigation of candidate genes associated with the salinity stress response and the development of salt-tolerant rice varieties.

## Supporting information

S1 Fig Content of Na^+^, K^+^ and Na^+^/K^+^ ratio in the roots (a, b, c) and shoots (d, e, f) in different treatments and sampling times. For each combination of genotypes, treatments and sampling times, different letters indicate a significant difference based on Duncan’s multiple range test (P ≤ 0.05).
(TIF)

S2 Fig Number of DEGs in 32 comparative combinations using 16 transcriptome samples. R: root, S: shoot, CT: control, SS: salt stress, 6 h: 6-hour timepoint, 54 h: 54-hour timepoint, CSR28: salt-tolerant genotype, IR28: salt-sensitive genotype.
(TIF)

S3 Fig Number of DEGs between (a) Organs, (b) Treatments, (c) Genotypes and (d) Timepoints. (TIF)

S4 Fig (a) Identification and grouping of TF families in the DEGs in response to salinity stress (comparison of control and salinity samples), (b) Heatmap for 60 differential genes belonging to the MYB transcription family. Values are based on log2 fold change. Red and green colors indicate increase and decrease of expression in response to salinity, respectively.
(TIF)

S5 Fig Part of GO functional sub term network at biological process level for salt-specific genes in the roots at 54-hour timepoint. (**TIF**)

S6 Fig Heatmap for highly expressed genes involved in biosynthesis of secondary metabolites using specific genes of salinity stress at seedling stage. Overexpression (high expression of genes as log2 FC ≥ 3 or ≤ -3) in tolerant cultivar CSR28 and sensitive cultivar IR28 are represented by green and red colors, respectively.
(TIF)

S7 Fig (a) Comparison of expression values of salinity-responsive genes in two approaches, RNA-Seq and qRT-PCR, (b) Correlation between RNA-Seq and qRT-PCR expression data. (**TIF**)

S8 Fig Protein-protein interaction (PPI) network analysis for up-regulated salt-specific genes in the roots of salt-sensitive IR28 at 54-hour timepoint. Top 25 hub genes are indicated by color intensity. Red nodes display highly dens interactions with other proteins.
(TIF)

S9 Fig GO enrichment analysis of 50 hub genes in the roots at 54-hour timepoint. BP: Biological process, MF: Molecular function, CC: Cellular component, CSR28: salt-tolerant genotype, IR28: salt-sensitive genotype.
(TIF)

S10 Fig Functional validation experiment of six hub genes encoding plastid RPs in response to salinity stress. No significant relationship was shown between dry weight and expression value of the hub genes.
(TIF)

S11 Fig Carotenoid biosynthesis pathway for salt-specific genes in the roots at 54-hour timepoint. The genes encoding enzymes are shown as red stars.
(TIF)

S1 Table ANOVA results for Na+, K+ and Na+/ K+ ratio in roots and shoots. (**DOCX**)

S2 Table RNA-Seq statistics. (**DOCX**)

S3 Table Significant (P-value cutoff ≤ 0.05) enriched terms of GO biological process (BP) for salt-responsive genes from comparison between the genotypes under salt-specific condition in different organs and timepoints. (**DOCX**)

S4 Table GO enrichment analysis of the hub genes in CSR28. (**XLS**)

S5 Table GO enrichment analysis of the hub genes in IR28. (**XLS**)

S6 Table KEGG enrichment analysis of the hub genes in CSR28. (**XLS**)

S7 Table Gene-specific primers used in qRT-PCR analysis for validation of RNA-Seq results. (**XLS**)

S8 Table Rice genotypes with various salinity tolerance used for functional validation of hub genes. (**XLS**)

S9 Table Gene-specific primers used in qRT-PCR analysis for functional validation of hub genes. (**DOCX**)
