# Supplementary figures and images for: Transcriptome and network analysis pinpoint ABA and plastid ribosomal proteins as main contributors to salinity tolerance in the rice variety, CSR28
Source: PLoS One. 2025 Apr 17;20(4):e0321181. doi: 10.1371/journal.pone.0321181 (PMC12005493; doi:10.1371/journal.pone.0321181)

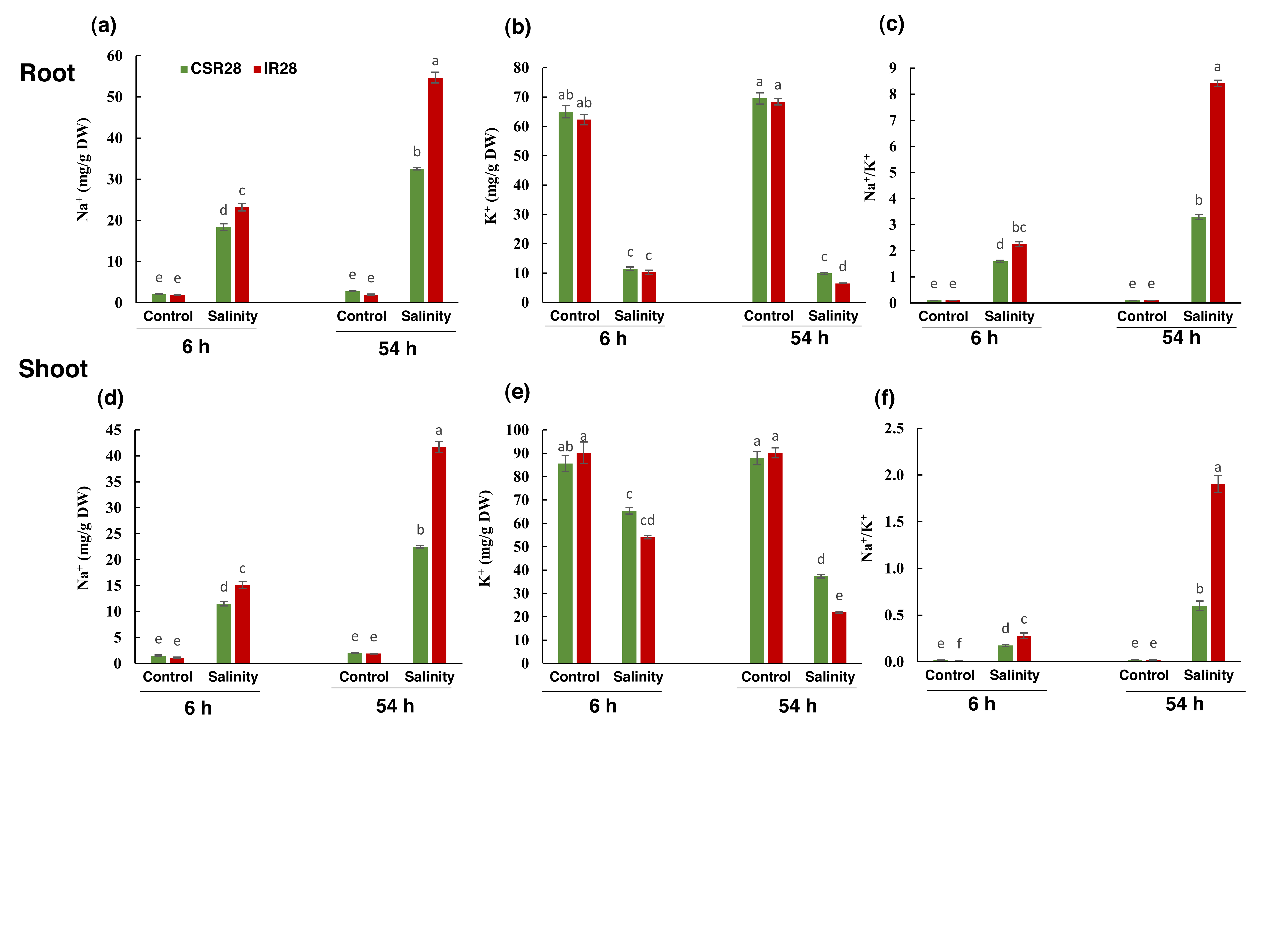

Supplement: S1 Fig — For each combination of genotypes, treatments and sampling times, different letters indicate a significant difference based on Duncan’s multiple range test (P ≤ 0.05). (TIF) [file pone.0321181.s001.tif]

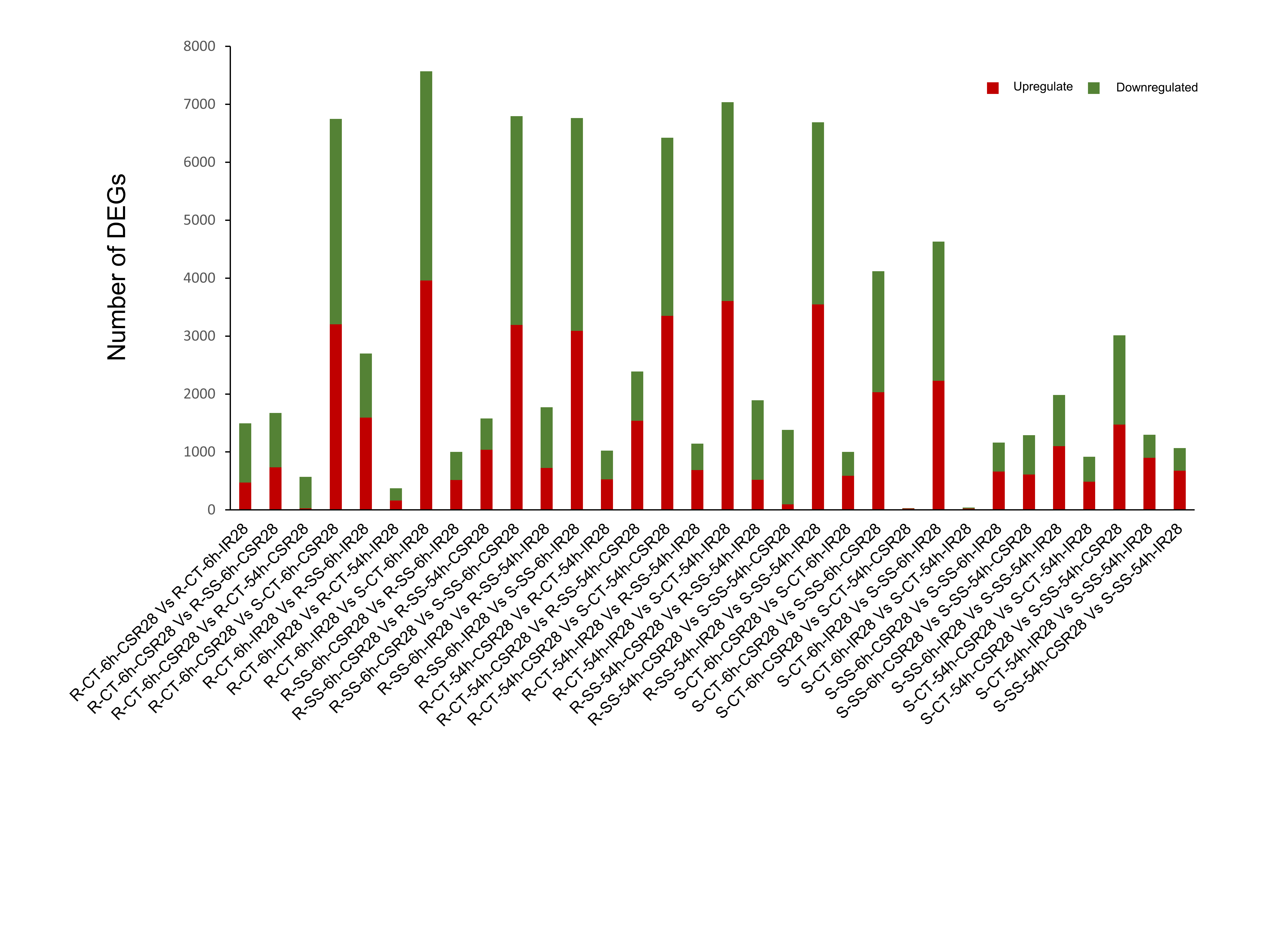

Supplement: S2 Fig — R: root, S: shoot, CT: control, SS: salt stress, 6 h: 6-hour timepoint, 54 h: 54-hour timepoint, CSR28: salt-tolerant genotype, IR28: salt-sensitive genotype. (TIF) [file pone.0321181.s002.tif]

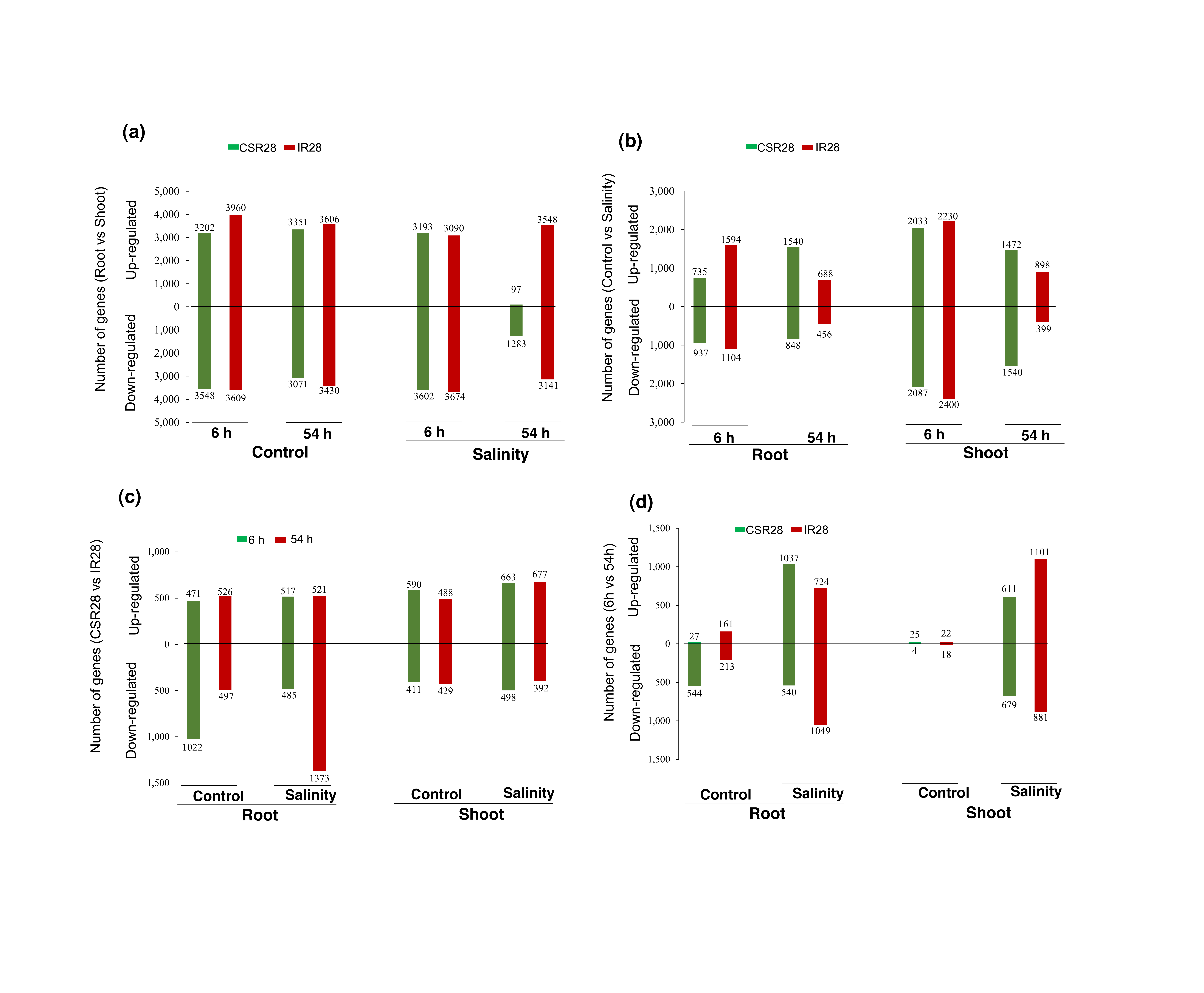

Supplement: S3 Fig — (TIF) [file pone.0321181.s003.tif]

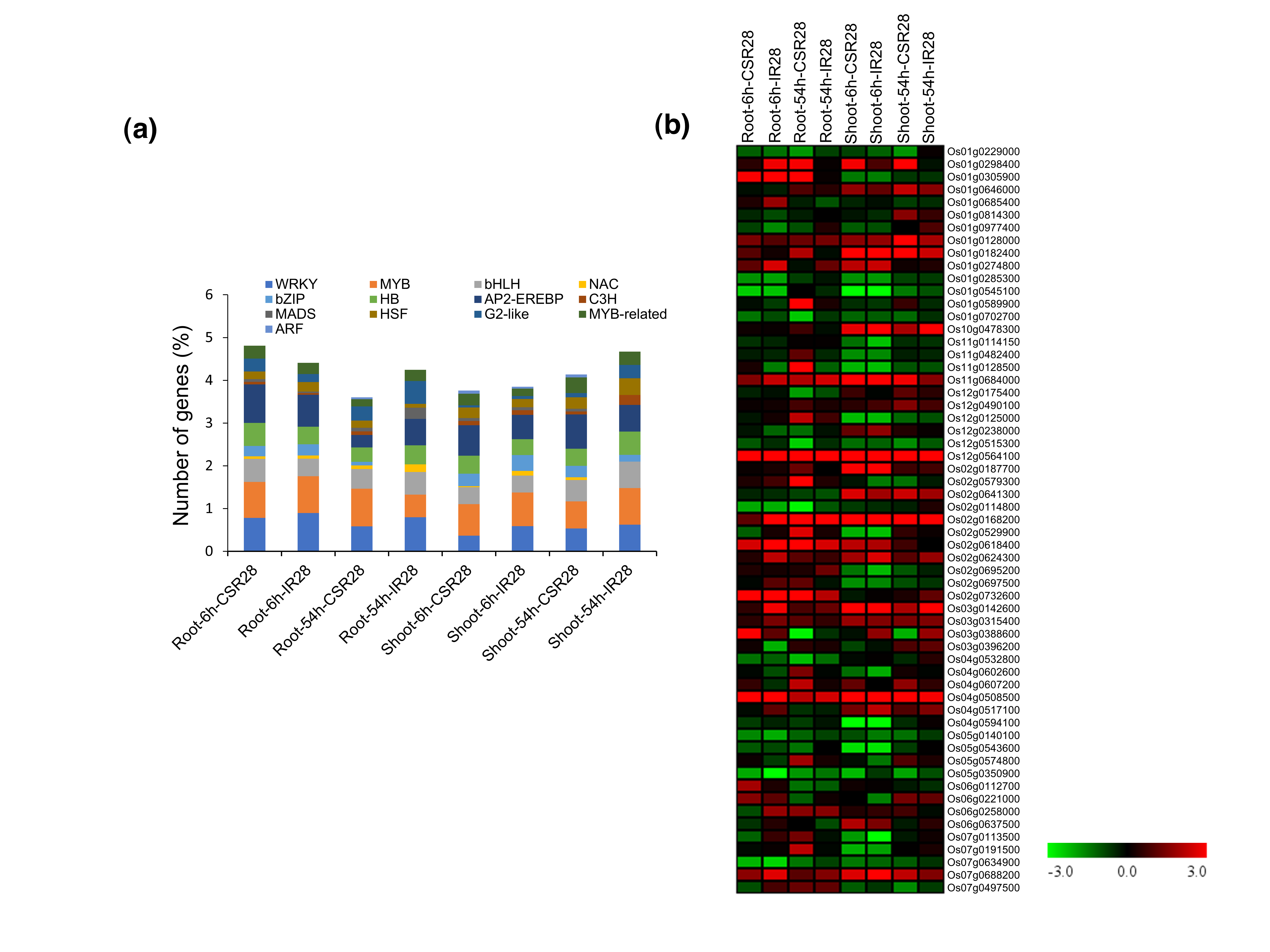

Supplement: S4 Fig — Values are based on log2 fold change. Red and green colors indicate increase and decrease of expression in response to salinity, respectively. (TIF) [file pone.0321181.s004.tif]

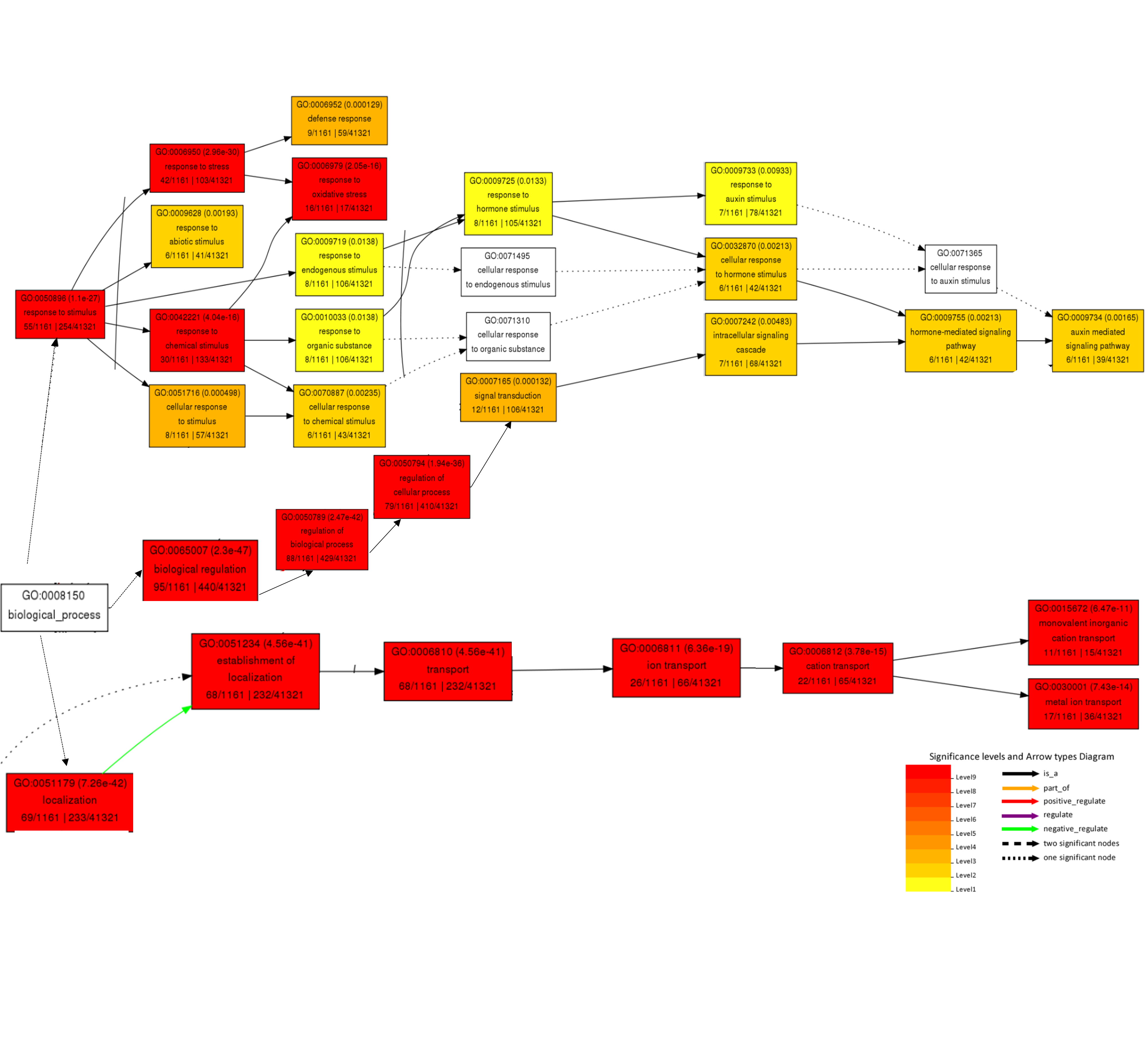

Supplement: S5 Fig — (TIF) [file pone.0321181.s005.tif]

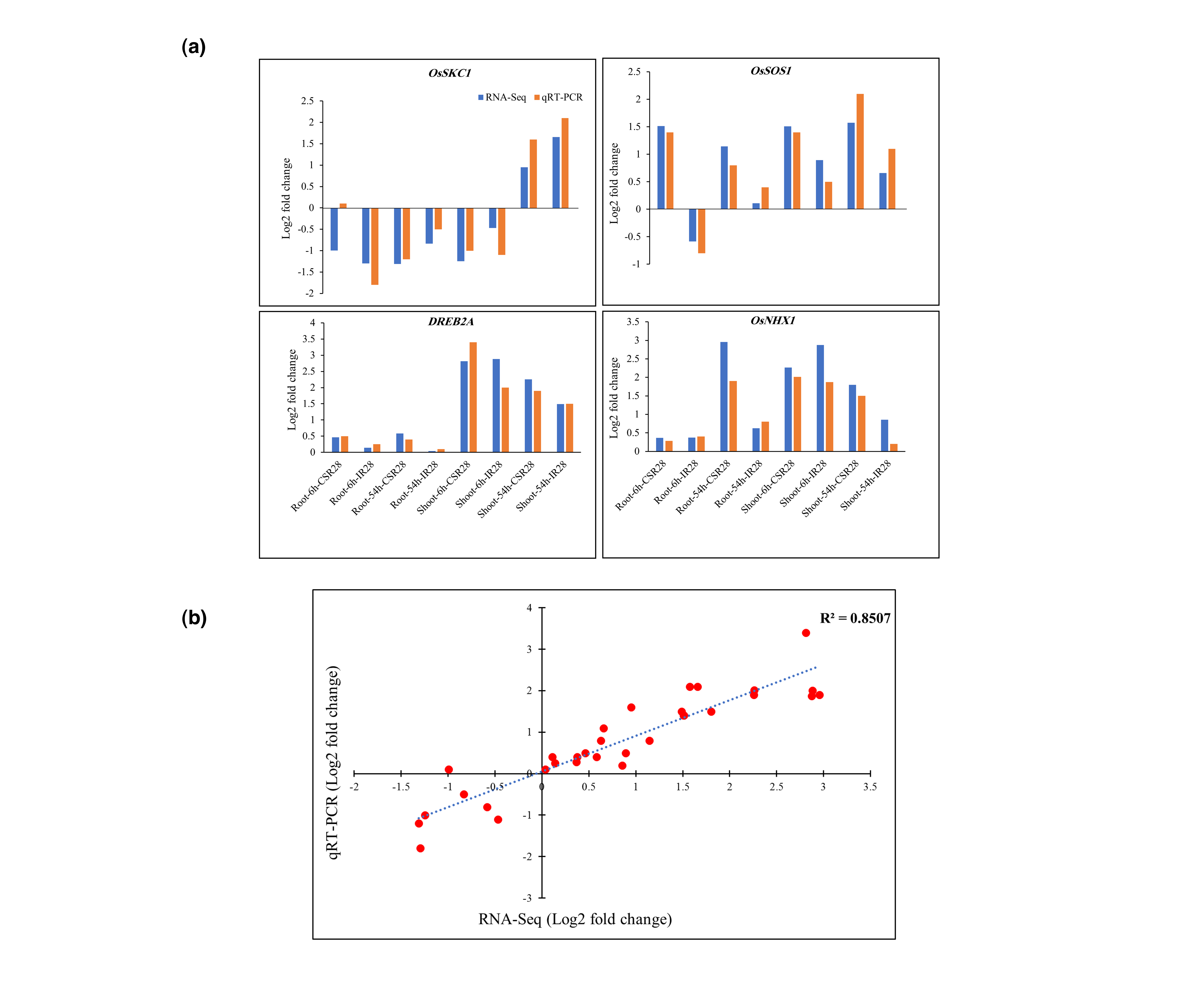

Supplement: S7 Fig — (TIF) [file pone.0321181.s007.tif]

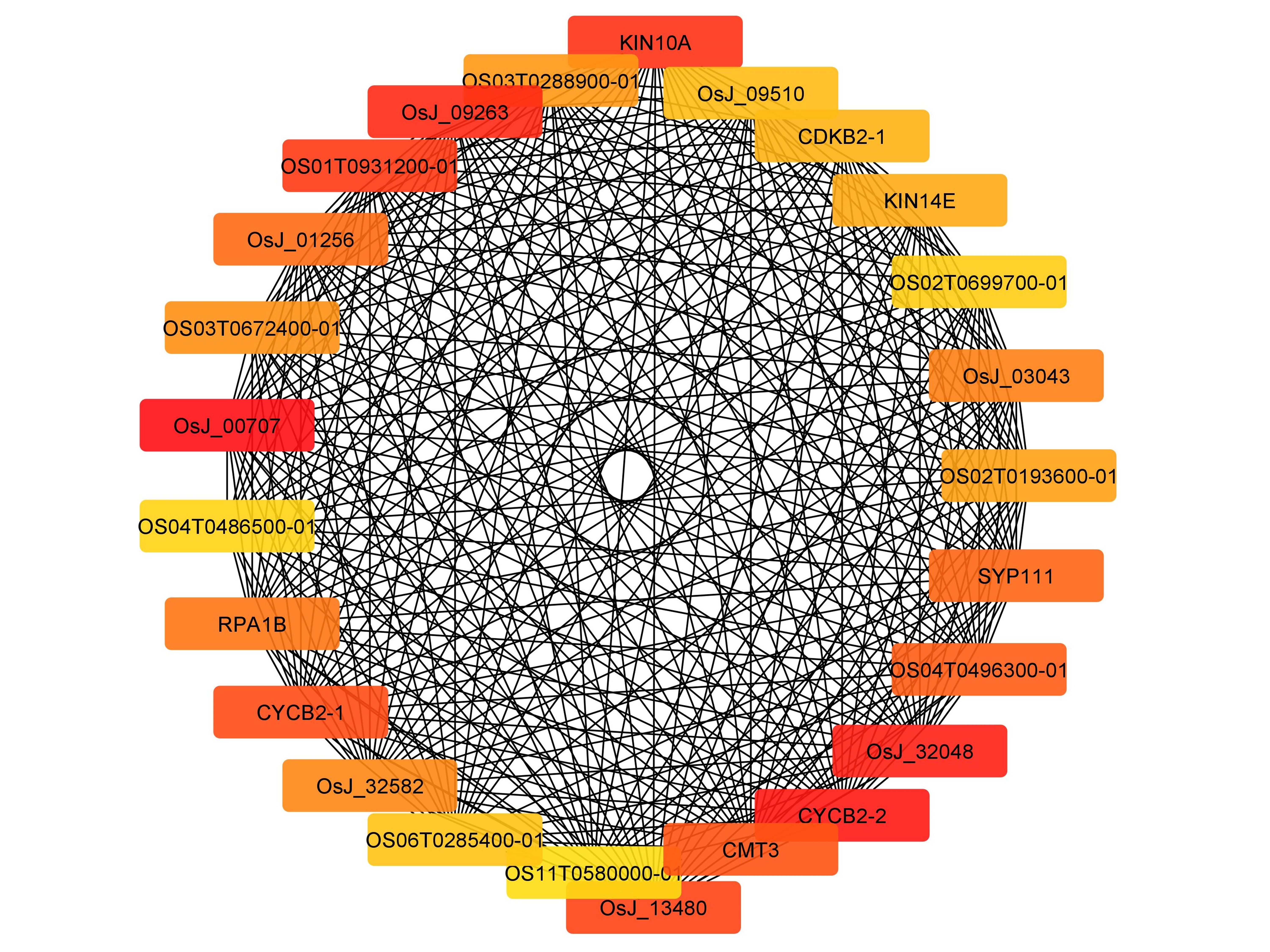

Supplement: S8 Fig — Top 25 hub genes are indicated by color intensity. Red nodes display highly dens interactions with other proteins. (TIF) [file pone.0321181.s008.tif]

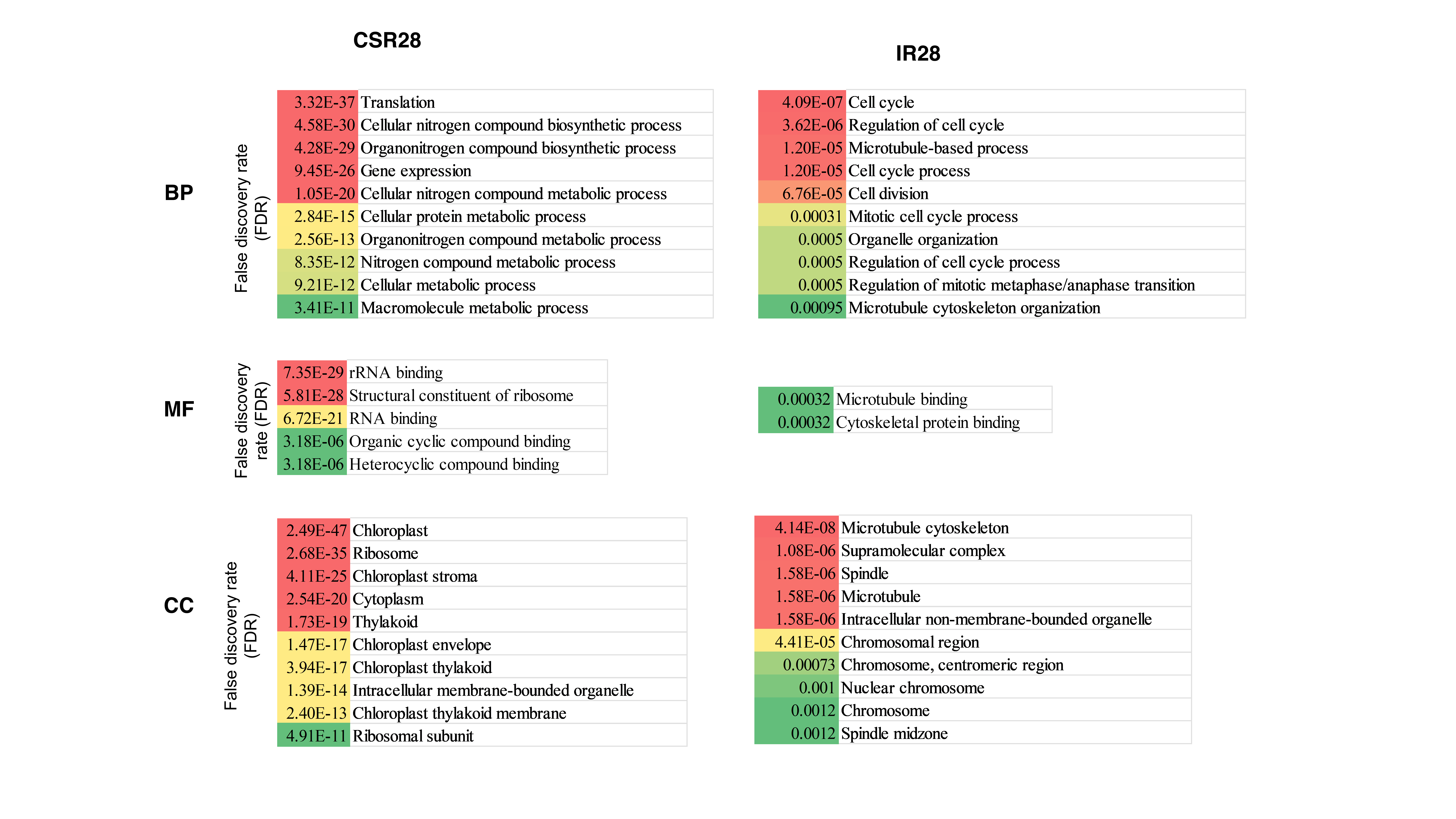

Supplement: S9 Fig — BP: Biological process, MF: Molecular function, CC: Cellular component, CSR28: salt-tolerant genotype, IR28: salt-sensitive genotype. (TIF) [file pone.0321181.s009.tif]

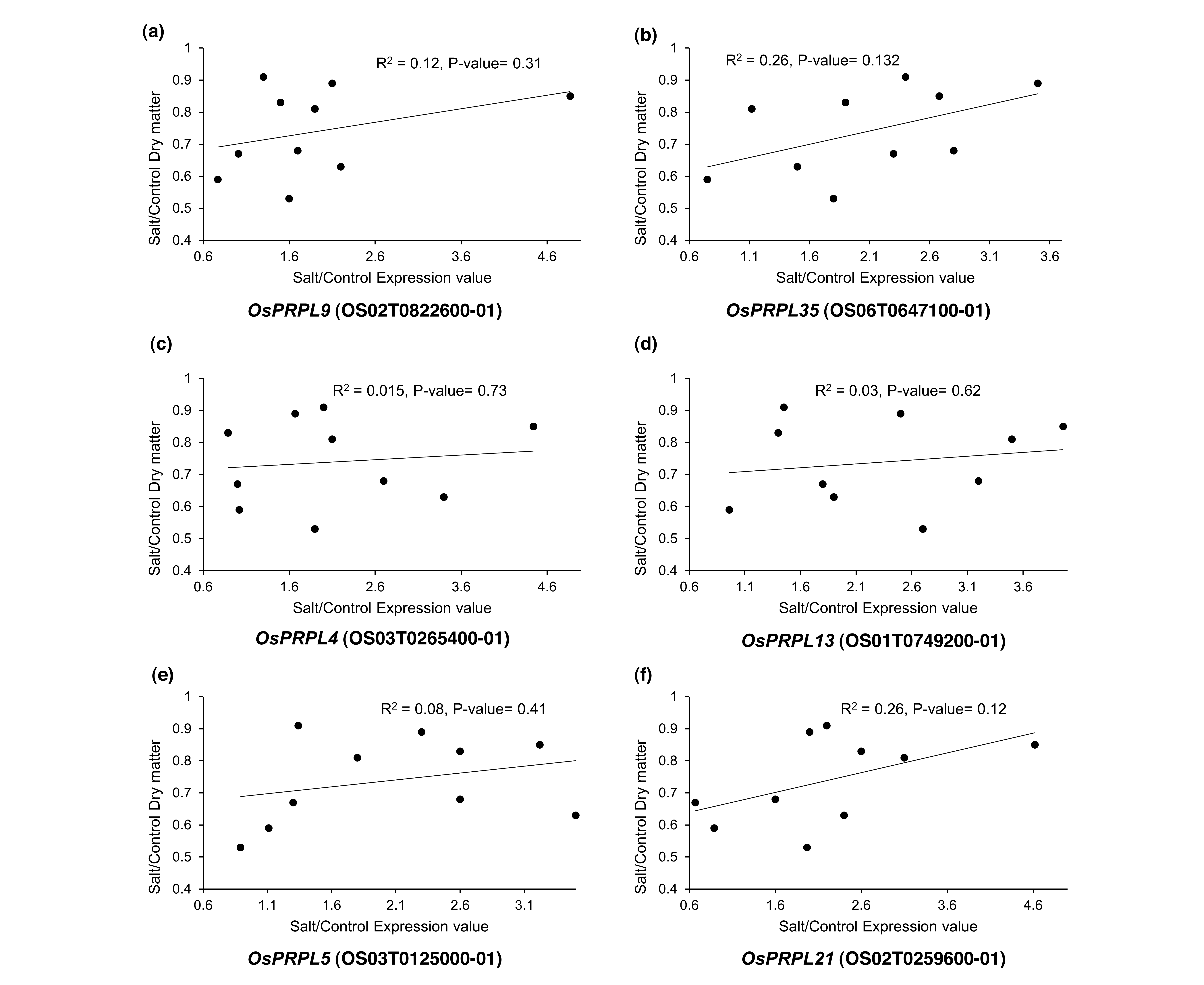

Supplement: S10 Fig — No significant relationship was shown between dry weight and expression value of the hub genes. (TIF) [file pone.0321181.s010.tif]

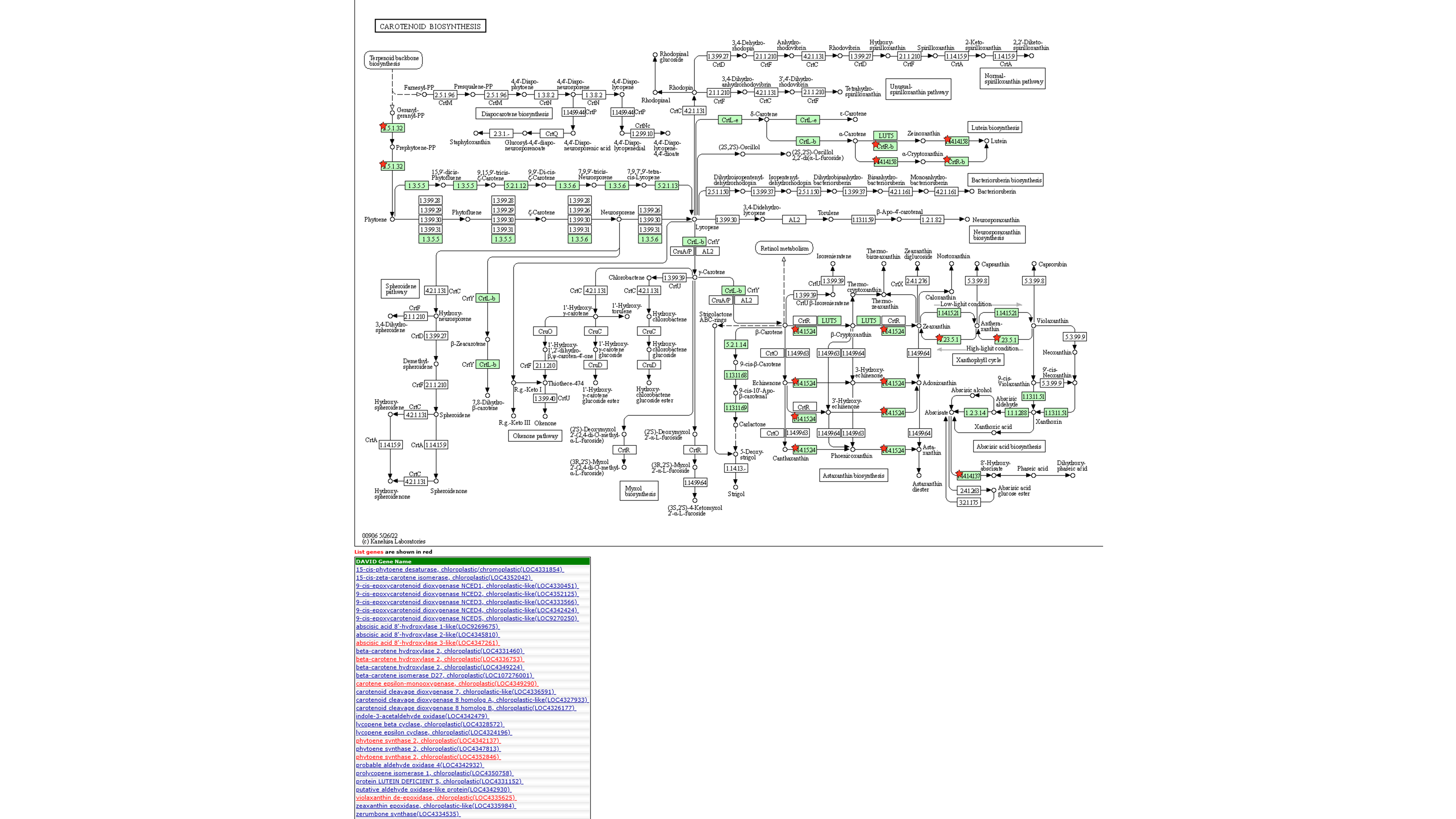

Supplement: S11 Fig — The genes encoding enzymes are shown as red stars. (TIF) [file pone.0321181.s011.tif]
