# Supplementary material for: Transcriptome and network analysis pinpoint ABA and plastid ribosomal proteins as main contributors to salinity tolerance in the rice variety, CSR28
Source: PLoS One. 2025 Apr 17;20(4):e0321181. doi: 10.1371/journal.pone.0321181 (PMC12005493; doi:10.1371/journal.pone.0321181)
Supplement: S1 Table — (DOCX) [file pone.0321181.s012.docx]

| Gene name | Gene ID | Primer Sequence (5’-3’) | Efficiency |
| --- | --- | --- | --- |
| *OsSOS1* | Os12g0641100 | F- CTCCGTGCTCATAGAATCGC  R- ATACTCACTCAAGTGGGTCAATACC | 0.93 |
| *DREB2A* | Os01g0165000 | F- AGATTGCTCCGTGCAAGTG  R- CTGGAGCTTCTGGTTTTGCT | 0.97 |
| *OsNHX1* | Os07g0666900 | F- GTTCAAGAGTTACAACAAAGCACG  R- CAGCGGGAATACAAAAGCAG | 1.01 |
| *OsHKT1;5 (*SKC1*)* | Os01g0307500 | F- TGCCACCTTACACCACTTTCG  R- TGCCATACGCACTGATAACCTC | 0.94 |
| *OsEF1a* | Os03g0177500 | F- CAACATTGTGGTCATTGGCC  R- GCAGTAGTACTTGGTGGTCT | 0.97 |

**Table S1** Gene-specific primers used in qRT-PCR analysis for validation of RNA-Seq results
