# Supplementary material for: Transcriptome and network analysis pinpoint ABA and plastid ribosomal proteins as main contributors to salinity tolerance in the rice variety, CSR28
Source: PLoS One. 2025 Apr 17;20(4):e0321181. doi: 10.1371/journal.pone.0321181 (PMC12005493; doi:10.1371/journal.pone.0321181)
Supplement: S2 Table — (DOCX) [file pone.0321181.s013.docx]

**Table S2** Rice genotypes with various salinity tolerance used for functional validation of hub genes

| Genotypes | Origin | Sensitivity degree |
| --- | --- | --- |
| Pokkali | India | Tolerant |
| FL478 | IRRI | Tolerant |
| Nano Bokra | India | Tolerant |
| IR651 | IRRI | Tolerant |
| CSR28 | India | Tolerant |
| Hashemi | Iran | Sensitive |
| Hasani | Iran | Semi-tolerant |
| Shirudi | Iran | Semi-tolerant |
| IR28 | IRRI | Sensitive |
| IR29 | IRRI | Sensitive |
