# Supplementary material for: Transcriptome and network analysis pinpoint ABA and plastid ribosomal proteins as main contributors to salinity tolerance in the rice variety, CSR28
Source: PLoS One. 2025 Apr 17;20(4):e0321181. doi: 10.1371/journal.pone.0321181 (PMC12005493; doi:10.1371/journal.pone.0321181)
Supplement: S3 Table — (DOCX) [file pone.0321181.s014.docx]

**Table S3** Gene-specific primers used in qRT-PCR analysis for functional validation of hub genes

| Gene symbol | Transcript ID | Primer Sequence (5’-3’) | Efficiency |
| --- | --- | --- | --- |
| *OsPRPL9* | OS02T0822600-01 | F-GTTCTACCGCAACTTCCTTC  R- CCTCTTCTTTTACCCGCTTC | 0.98 |
| *OsPRPL17* | OS03T0815400-01 | F-GTGTTCCTAAACTTAGCAGACC  R- TCTTGTGAAGAGACCCATCC | 0.93 |
| *OsPRPS9* | OS03T0769100-01 | F-TCATCAACTTCCGCGATGCC  R- GACCAGCCTTCTTCCTTTCAAC | 1.02 |
| *OsPRPL11* | OS03T0122200-01 | F-CCTTTATCTTGAAGACCCCTCC  R- CCATATTAGCAGCAGTGCC | 0.96 |
| *OsPRPL35* | OS06T0647100-01 | F-GTACAAGATGAAGACCCACAAG  R- CACGTTGTTGTAGTCGCTC | 1.03 |
| *OsPRPL4* | OS03T0265400-01 | F-ACTCTCAACCTATACGACATCC  R- CTTCCTCCTCACCATCATCC | 0.94 |
| *OsPRPL13* | OS01T0749200-01 | F-AGCAGAGCGAGAAGCAGAAG  R- ACCACAACAACAAAAGCCCC | 1.05 |
| *OsPRPL5* | OS03T0125000-01 | F-GTCGCCAGTTTCAAGATCC  R- CATTCCATTCTTCTTCCCACC | 0.99 |
| *OsPRPL21* | OS02T0259600-01 | F-CCTCCAAAACCCTAGCCTTC  R- CCTCCTCTTCTTCAACCTCC | 1.01 |
| *OsEF1a* | Os03g0177500 | F- CAACATTGTGGTCATTGGCC  R- GCAGTAGTACTTGGTGGTCT | 0.98 |
