# Supplementary material for: Transcriptome and network analysis pinpoint ABA and plastid ribosomal proteins as main contributors to salinity tolerance in the rice variety, CSR28
Source: PLoS One. 2025 Apr 17;20(4):e0321181. doi: 10.1371/journal.pone.0321181 (PMC12005493; doi:10.1371/journal.pone.0321181)
Supplement: S9 Table — (DOCX) [file pone.0321181.s020.docx]

**Table S9.** KEGG enrichment analysis of the hub genes in CSR28

| Category | ID | term | Number of genes | FDR | genes |
| --- | --- | --- | --- | --- | --- |
| KEGG | map03010 | Ribosome | 21 | 1.46E-25 | OsJ_02723, OsJ_03005, OsJ_03455, OsJ_06142, OS02T0652600-02, OS02T0822600-01, OS03T0122200-01, RPL5, OsJ_09949, OS03T0265400-01, OsJ_10397, OS03T0356300-01, OsJ_12269, OsJ_12590, rps9, OsJ_13095, RPL18, OsJ_16775, OsJ_18402, OsJ_22158, OsJ_22397 |
